# Effectiveness of Interventions to Support Carers of People With Dementia in Low‐ and Middle‐Income Countries: A Systematic Review and Meta‐Analysis

**DOI:** 10.1002/gps.70054

**Published:** 2025-02-27

**Authors:** Frank Chen, Zhiwei Hu, Quan Li, Xuan Zheng, Meizhi Li, Maximilian Salcher‐Konrad, Adelina Comas‐Herrera, Martin Knapp, Cheng Shi, The STRiDE consortium

**Affiliations:** ^1^ Department of Health Policy London School of Economics and Political Science London UK; ^2^ First Clinical Medical School Suzhou Medical College of Soochow University Suzhou China; ^3^ Department of Obstetrics and Gynecology The First Affiliated Hospital of Soochow University Suzhou China; ^4^ Department of Pulmonary and Critical Care Medicine The Second Xiangya Hospital of Central South University Changsha China; ^5^ Department of Medical Administration The Second Xiangya Hospital of Central South University Changsha China; ^6^ Care Policy and Evaluation Centre London School of Economics and Political Science London UK; ^7^ School of Graduate Studies and Institute of Policy Studies Lingnan University Hong Kong China

**Keywords:** caregiving, carer interventions, carer strain, carer support, dementia, effectiveness, LMICs, meta‐analysis, randomised controlled trials, systematic review

## Abstract

**Objectives:**

Family and other carers of people with dementia can potentially benefit from training and support to reduce the negative impacts of caregiving and prevent harm to care recipients. While interventions for carers in low‐ and middle‐income countries (LMICs) are emerging, their effectiveness is not well understood. Through a systematic review and meta‐analysis, the objective was to evaluate the effectiveness of interventions to support carers of people with dementia in improving the well‐being of carers and their care recipients in LMICs.

**Methods:**

This review, registered with PROSPERO (CRD42018106206), built on a systematic mapping of dementia interventions in LMICs under the Strengthening Responses to Dementia (STRiDE) project. It analysed evidence on interventions to support carers in these regions. Title and abstract screening, full‐text review, data extraction and risk of bias assessment were each conducted by two reviewers independently, with disagreements resolved through group discussion. Pairwise meta‐analyses were conducted, with robustness tested via leave‐one‐out analysis. Heterogeneity was explored using subgroup analysis, meta‐regression and MetaForest. Medline, Embase, Global Health and PsycINFO (via Ovid) and CINAHL (via EBSCO) databases were searched. We included randomised control trials focused on carer well‐being in LMICs, 2008–2022. Primary outcomes were perceived burden and depression; other health‐related quantitative outcomes were collected.

**Results:**

From 5228 records, 48 studies in English and Chinese were identified as eligible, reporting on 67 carer outcomes and 36 care recipient outcomes. Forty‐one studies were at high risk of bias. Meta‐analysis revealed statistically significant medium‐to‐large intervention effects on three key carer outcomes—perceived burden, depression, and anxiety—and on four major outcomes for people with dementia—neuropsychiatric symptoms, cognitive function, quality of life (QoL), and activities of daily living (ADL). These effects were larger than those typically observed in previous studies in high‐income countries (HICs).

**Conclusions:**

This review provides a comparative overview and summarises the characteristics of published interventions to support carers in LMICs. It reveals medium‐to‐large beneficial effects of the interventions on several key outcomes for carers and care recipients in LMICs. Future research employing more rigorous methodologies is recommended, particularly for broader and more diverse populations.

**Summary**

- Despite the significant increase in dementia research in low‐ and middle‐income countries (LMICs), evidence from high‐quality randomised controlled trials (RCTs) remained limited.
- Existing interventions in LMICs target one or more of three main objectives: improving knowledge about dementia, reducing care dependency, and enhancing carers' mental health.
- The interventions to support carers, in general, yielded statistically significant, medium‐to‐large effects on improving carers' perceived burden, depression, and other health indicators, with dyadic interventions for both people with dementia and carers being more effective.
- To scale up supportive interventions for a broader population of carers, high‐quality RCTs and culturally tailored approaches are urgently required to bolster the evidence base and enhance their effectiveness.

## 1 Introduction

Many people with dementia are supported by family and other formal or informal carers [1, 2, 3]. This can create a difficult balance between personal needs and care responsibilities, which often induces significant stress that affects the mental and physical well‐being of carers (sometimes called ‘caregivers’) [2, 3, 4]. Carers in low‐ and middle‐income countries (LMICs) encounter even greater challenges than those in high‐income countries (HICs) due to lower socioeconomic conditions and minimal social support [5, 6, 7, 8].

Interventions addressing negative outcomes of caregiving can be effective, but these interventions, such as Resources for Enhancing Alzheimer's Caregiver Health (REACH) [9, 10, 11] and Strategies for Relatives [12], are primarily conducted in some HICs. While recent systematic reviews support their effectiveness [13, 14, 15], evidence from LMICs, where two‐thirds of people with dementia live, is sparse. Due to different socioeconomic, cultural and epidemiological contexts, interventions from HICs may not be directly applicable to LMICs [16, 17, 18].

To address the unique challenges in LMICs, original and adapted interventions to support carers have been developed and sometimes trialled in LMICs. This study aimed to quantify the effectiveness of these interventions in LMICs and to summarise their designs [19], providing insights to guide the design and implementation of the interventions, especially in resource‐limited settings.

## 2 Methods

### 2.1 Search Strategy and Eligibility Criteria

This review built on the Strengthening Responses to Dementia in Developing Countries (STRiDE) program's systematic mapping of dementia intervention studies, which identified trials of interventions for people living with dementia or their carers in LMICs published between 2008–2018 [20].

We performed two additional database searches of Medline, Embase, Global Health and PsyclNFO via Ovid, plus CINHL via EBSCO to include studies published 2019–2022 on 21 September 2022 and 25 February 2023, using identical syntax to ensure continuity, with an additional term ‘careg*’ to focus on interventions to support carers (Table S2). To complement database searches, we manually reviewed lists of studies included in three recently published systematic reviews of interventions that involved carers of people with dementia [15, 21, 22].

We included studies published 2008−2022 meeting our inclusion criteria, shown below in the PICOS (Participants, Intervention, Comparator, Outcomes, Study design) format:

- Population: People with dementia and their carers living in LMICs, as defined by the Organisation for Economic Co‐operation and Development.
- Interventions: Interventions involving people with dementia and their carers or carers alone.
- Comparators: Any comparator, but only ‘no active intervention’ used for pairwise meta‐analysis.
- Outcomes: We considered perceived burden and depression as our primary outcomes because they were the most frequently measured outcomes in interventional studies targeting the well‐being of carers of people with dementia [23, 24, 25]. Secondary outcomes were any other quantitative outcome assessing the health of people with dementia and their carers [25].
- Study design: Randomised controlled trials (RCTs).

Detailed exclusion criteria were set out in our previously published protocol [26]. Research not conducted in LMICs, not involving people with dementia or their carers, not evaluating an intervention, not published in a language spoken by a member of our study team or the original STRiDE evidence review group (comprising 51 researchers fluent in 15 languages in total) were excluded.

### 2.2 Quality Assessment

The risk of bias was assessed with the Cochrane risk of bias tool version 2 (RoB2) on an individual study level and visualised with the R package ‘robvis’ [27, 28]. Each study was evaluated by at least two reviewers, with disagreement settled through group consensus. Publication bias was assessed through funnel plots and Egger's test only if the outcome was measured in more than 10 studies to ensure sufficient power [29, 30]. Industry bias was evaluated by checking funding sources. In each meta‐analysis, we also compared fixed‐ and random‐effects estimates to evaluate small‐study effects [31, 32].

### 2.3 Data Extraction

Two independent reviewers extracted and recorded data, resolving disagreements through group discussions. Recorded data included mean and standard deviation (SD) of outcomes pre‐ and post‐intervention, participant numbers per arm, trial settings, randomisation methods, measurement timing and funding sources. Participant demographics encompassed mean age and female proportion, with additional details on dementia type and stage where available. Intervention details included content, duration, frequency and delivery mode.

### 2.4 Qualitative Synthesis and Intervention Classification

We conducted qualitative analysis to assess the comparability of study designs, participants, intervention contents and outcome measures. The identified interventions were grouped based on similarities and differences between them [33]. Specifically, we categorised interventions according to their target populations (people with dementia, their carers, or both), the techniques employed, and their stated rationale or theories of change (ToCs) [34]. This grouping aimed to reduce within‐group heterogeneity in meta‐analysis due to variation in intervention content. We also analysed the intervention content to determine potential effectiveness beyond their original settings, considering the limited resources for dementia care in LMICs [35].

### 2.5 Quantitative Analysis

Aiming to obtain a summary effect estimate of treatment effects of any intervention compared to no (active) intervention, we performed pairwise meta‐analysis, with all interventions grouped together, followed by subgroup analysis and meta‐regression, using the R package ‘meta’ [36]. We converted outcome data into Hedge's g, using the R package ‘esc’ [37, 38]. To ensure sufficient statistical power, we meta‐analysed outcomes measured in at least five trials [39]. In addition to 95% confidence intervals (CIs), we also calculated 95% prediction intervals (PIs), which show the uncertainty expected in the outcome measure if a new study was included in the meta‐analysis, and so predict the intervention effects seen in future trials.

Heterogeneity across studies was evaluated with *I* ^2^ statistics and Cochran's *Q* test [40]. We used the R package ‘dmetar’ to perform influence diagnostics [41] and leave‐one‐out analysis [42], to identify potential influential cases, or the extreme results that significantly altered meta‐analytic results once included in the analysis. Subgroup analysis and meta‐regression were performed for any outcome measured in at least 10 trials to ensure sufficient power [13, 14, 15]. To further explore potential sources of heterogeneity, we used the R package ‘MetaForest’, which provides a robust, machine learning‐based approach to measure variable importance, even when the sample size is small.

## 3 Results

### 3.1 Study Selection

**FIGURE 1:**
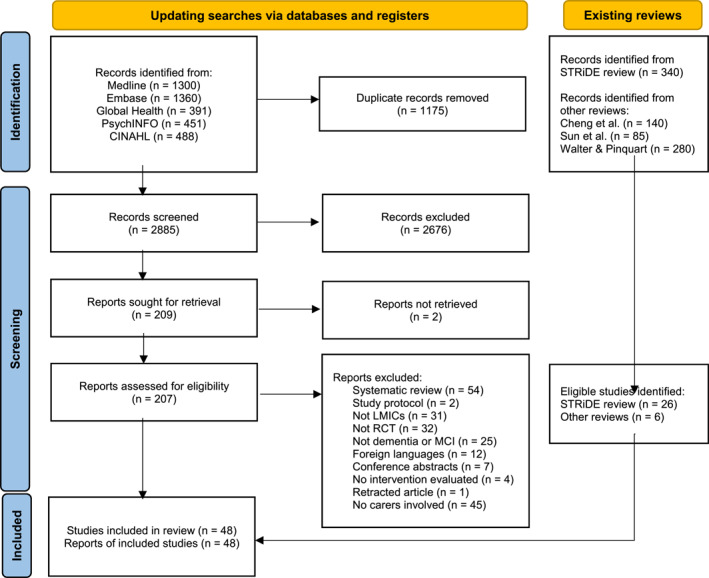
Flow chart of study selection. Adapted from PRISMA 2020 Statement. The STRiDE consortium's languages include Arabic, Bengali, Chinese, English, French, Hindi, Kannada, German, Bahasa Indonesia, Portuguese, Romanian, Spanish, Tamil, Telugu, and Turkish, encompassing seven of the 10 most widely spoken languages globally. No language restrictions were applied during the database search. Full texts were retrieved after screening English‐language titles and abstracts, even if the main text was in another language. However, full‐text screening was only conducted for papers written in one of the 15 specified languages. 12 studies in other languages, which could not be evaluated against the inclusion criteria, were excluded.

Forty‐eight studies from LMICs published between 2008 and 2022 were included (Figure 1), of which 26 studies had been screened from the previous STRiDE review. We also identified seven additional studies from three recently published meta‐analyses [15, 21, 22]. Of all included studies, 38 were in English and 10 in Chinese. No studies were found that were published in the other 13 languages used by the STRiDE consortium, as listed in our published protocol [26].

### 3.2 Study Characteristics

Study characteristics are summarised in Table 1.

**TABLE 1:** Selected characteristics of included studies.

Study ID^a^	Study characteristics					Intervention characteristics					
	Country	Funding^b^	Setting		Randomisation	Descriptive name	Participants^c^	Control	Intervention type	Mode(s)	Frequency
Arango‐Lasprilla 2014	Colombia	Self	Urban	Lecture hall	Randomised by carer	Educational program	Informal carers	Cognitive‐behavioural intervention	Training for dementia and self care	Group sessions	Weekly
						Coping with frustration class	Informal carers	Basic psychoeducation	Thematic skill training	Group sessions	Weekly
Aslan 2022	Turkey	Self	Urban	Tertiary hospital	Randomised by carer	PLST‐based intervention	Dyad	Routine care	Training for BPSD management	Group sessions	Weekly
Baruah 2021	India	NGO	Virtual	Website	Randomised by carer	The interactive iSupport program	Informal carers	Reading materials	Guided self‐study	Interactive online platform	N/A
Chen 2020	China	Govt.	Urban	Instant messaging	Randomised by patient	Extended nursing program	Dyad, nurses	Routine care	Comprehensive care for people with dementia	Online, group sessions	> Weekly
Danucalov 2013	Brazil	NGO	Urban	Home	Randomised by carer	Yoga and comparison meditation program	Informal carers	Routine care	Body‐mind intervention	Group and online sessions	> Weekly
Dias 2008	India	IGO	Semi‐urban	Home	Randomised by dyad	Helping carers to care intervention	Informal carers	Reduced training, waitlist	Training for dementia and self care	Home visits, in clinic	Weekly to monthly
Duru Asiret 2021	Turkey	Univ.	Urban	Tertiary hospital	Randomised by patient	PLST‐based intervention	Dyad	Educational materials	Training for BPSD management	Group sessions, home visits, phone calls	Weekly to monthly
Ghaffari 2019	Iran	Univ.	Urban	Tertiary hospital	Block‐randomised by carer	Resilience education program	Informal carers	Routine care	Thematic skill training	Group sessions	Weekly
Gok Ugur 2018	Turkey	Univ.	Urban	Home	Randomised by patient	Music therapy	Informal carers	Routine care	Music therapy for dementia patients	Individual sessions	> Weekly
Govindakumari 2020	India	Self	Semi‐urban	Home	Randomised by patient	Cognitive training program	Person with dementia	No information	Cognitive and functional training for dementia patients	Individual sessions	> Weekly
Guerra 2011	Peru	NGO	Urban	Home	Stratified permuted block randomisation by carer	Helping carers to care intervention	Informal carers	Waitlist	Training for dementia and self care	Home visits	Weekly
He 2012	China	Govt.	Urban	Tertiary hospital	Randomised by patient	Reminiscence therapy	Person with dementia	Routine care	Cognitive and functional training for dementia patients	Group sessions, individual sessions	Weekly to monthly
Heydari 2017	Iran	Univ.	Urban	Tertiary hospital	Randomised by carer	Problem oriented coping strategies training	Informal carers	Reduced training	Thematic skill training	Group sessions	Weekly
Hinton 2020	Vietnam	Govt.	Semi‐urban	Community	Cluster‐randomised by community	REACH VN	Informal carers	Reduced training	Training for dementia and self care	Home visits	Weekly
Jahani 2022	Iran	Univ.	Virtual	Instant messaging	Block‐randomised by carer	Compassion‐based program	Informal carers	No information	Thematic skill training	Online sessions, phone interviews	> Weekly
Jiang 2012	China	N/A	Urban	Tertiary hospital	Randomised by patient	Community‐family care interventions	Dyad, nurses	Routine care	Comprehensive care for people with dementia	In clinic, home visits, phone interviews	Weekly
Kamkhagi 2015	Brazil	NGO, pvtd., govt.	Urban	Tertiary hospital	Randomised by carer	Bodily awareness therapy	Informal carers	Psychodynamic group therapy	Body‐mind intervention	Group sessions	Weekly
						Psychodynamic group therapy	Informal carers	Bodily awareness therapy	Group therapy	Group sessions	Weekly
Liu 2017	China	Univ.	Urban	Community	Randomised by carer	Family visits of community nurses	Dyad, nurses	Routine care	Comprehensive care for people with dementia	Training for professional carers, home visits, telephone interview	Weekly
Lök 2017	Turkey	N/A	Urban	Home	Randomised by carer	“First you should get stronger” caregiving program	Informal carers	No information	Training for dementia and self care	Home visits	Weekly
Lök 2019	Turkey	N/A	Urban	Nursing home	Randomised by patient	Reminiscence therapy	People with dementia	Routine care	Cognitive and functional training for dementia patients	Group sessions	Weekly
Mahdavi 2017	Iran	Univ.	Urban	Lecture hall	Block‐randomised by carer	Spiritual group therapy	Informal carers	Routine care	Spiritual group therapy	Group sessions	Weekly
Novelli 2018	Brazil	Govt.	Urban	Home	Randomised by dyad	Tailored activity program	Dyad	Waitlist	Training for BPSD management	Home visits	Weekly to monthly
Oliveira 2018	Brazil	Govt., NGO	Urban	Home	Randomised by patient	Psychoeducation group sessions	Informal carers	Multiple interventions	Training for dementia and self care	Individual sessions	Weekly
						Tailored activity program	Dyad	Multiple interventions	Training for BPSD management	Individual sessions	Weekly
Oliveira 2021	Brazil	Govt., NGO	Urban	Tertiary hospital	Randomised by patient	Psychoeducation group sessions	Informal carers	Multiple interventions	Training for dementia and self care	Group sessions	Weekly to monthly
						Tailored activity program	Dyad	Multiple interventions	Training for BPSD management	Group sessions	Weekly to monthly
Pahlavanzadeh 2010	Iran	Univ.	Urban	Tertiary hospital	Randomised by carer	Family education program	Dyad	Routine care	Training for BPSD management	Group sessions	Weekly
Pan 2019	China	Govt.	Urban	Lecture hall	Block‐randomised by carer	Nurse‐led cognitive‐behavioural intervention	Informal carers	Unguided group sessions	Thematic skill training	Individual sessions, phone interviews	Monthly
Pankong 2018	Thailand	N/A	Semi‐urban	Community	Randomised by carer	Enhancing positive aspects of caregiving program	Informal carers	Routine care	Thematic skill training	Group sessions	Weekly
Serrani Azcurra 2012	Argentina	N/A	Urban	Nursing home	Randomised by patient	Reminiscence therapy	Person with dementia	Unguided group sessions	Cognitive and functional training for dementia patients	Group sessions	Weekly to monthly
Shata 2017	Egypt	Self	Urban	Tertiary hospital	Randomised by dyad	Group psychosocial intervention program	Informal carers	No information	Training for dementia and self care	Group sessions	Weekly
Söylemez 2016	Turkey	Univ.	Urban	Home	Randomised by dyad	PLST‐based intervention	Dyad	Routine care education, educational materials	Training for BPSD management	Home visits, phone interviews	Weekly to monthly
SU 2012	China	N/A	Urban	Tertiary hospital	Randomised by patient	Comprehensive nursing intervention	Dyad, nurses	Routine care	Comprehensive care for people with dementia	In clinic, home visits, phone interviews	Weekly to monthly
Sun 2010	China	N/A	Urban	Community	Randomised by patient	Nursing intervention	Dyad, nurses	Routine care	Comprehensive care for people with dementia	In clinic, home visits, phone interviews, group sessions	Weekly
Tan 2010	China	N/A	Urban	Community	Randomised by patient	Early family nursing intervention	Dyad, nurses	Routine care	Comprehensive care for people with dementia	Home visits, phone interviews, group sessions	Weekly
Tawfik 2021	Egypt	Self	Urban	Tertiary hospital	Randomised by carer	Psychoeducational program	Informal carers	Routine care	Training for dementia and self care	Group sessions	Weekly
Turten kaymaz 2017	Turkey	N/A	Urban	Home	Randomised by patient	Aromatherapy	Informal carers	Routine care	Aromatherapy	Individual sessions	> Weekly
Uyar 2019	Turkey	N/A	Urban	Home	Randomised by dyad	Multi‐component intervention program	Informal carers	Routine care	Training for dementia and self care	Group sessions	Weekly to monthly
Wang 2010	China	Govt.	Urban	Community	Randomised by patient	Community nursing intervention	Dyad, nurses	Routine care	Comprehensive care for people with dementia	Home visits, phone interviews, group sessions	Weekly
Wang 2012	China	N/A	Urban	Community	Randomised by carer	Mutual support group	Informal carers	Routine care	Group therapy	Group sessions	Weekly to monthly
Wang 2014	China	N/A	Urban	Home	Randomised by patient	Home‐based care intervention	Dyad, nurses	Routine care	Comprehensive care for people with dementia	Home visits	Monthly
Wang 2017c	China	Univ.	Urban	Community	Cluster‐randomised by community	Dementia‐specific training	Nurses	Irrelevant training	Nursing education	Group sessions, online sessions	Weekly to monthly
Wang 2017e	China	Govt.	Virtual	Instant messaging	Cluster‐randomised by community	Dementia education and knowledge translation program	Nurses	Waitlist	Nursing education	Group sessions	Weekly
Wang 2021	China	N/A	Urban	Homes	Randomised by carer	Professional‐guided bibliotherapy	Informal carers	Routine care	Guided self‐study	Individual sessions, phone interviews, home visits	Weekly
Xu 2022	China	N/A	Virtual	Telephone	Randomised by carer	Telephone‐based behavioural activation program	Informal carers	Routine care	Thematic skill training	Phone interviews	> Weekly
Yang 2017	China	Govt.	Urban	Community	Cluster‐randomised by community	Family medical intervention model	Dyad, nurses	General community health education	Comprehensive care for people with dementia	Group sessions	Weekly
Yang 2021	China	Govt.	Urban	Nursing home	Randomised by patient	Comprehensive intervention on person with dementia life quality	Dyad, nurses	Routine care	Comprehensive care for people with dementia	Group sessions, individual sessions	Weekly to monthly
Zarepour 2020	Iran	Univ.	Urban	Lecture hall	Block‐randomised by carer	Educational intervention on the anxiety of family carers	Informal carers	Routine care	Training for dementia and self care	Group sessions, phone interviews	> Weekly
Zhang 2021	China	Govt.	Urban	Nursing home	Randomised by patient	Carer training program on oral hygiene	Dyad	Reduced training	Dyadic intervention for oral hygiene	Individual sessions	Weekly to monthly
Zhao 2010	China	N/A	Urban	Home	Randomised by patient	Family nursing intervention	Dyad, nurses	Routine care	Comprehensive care for people with dementia	Home visits, phone interviews, group sessions	Weekly

^a^ Please find the detailed citation of each study in Table S9. Participant characteristics are available in Table S4.

^b^ Funding sources include self‐funding (Self), non‐governmental organizations (NGOs), governments (Govt.), universities (Univ.), private companies (Priv.), and the World Health Organization (WHO). “N/A” indicates that data is not available.

^c^ Definitions of participants: Informal carers, often family members, provide care to individuals with whom they share a personal connection. Many interventional studies listed here focus on the dyad of the person with dementia and their informal carer. In some cases, nurses—who are a common type of professional carer—are also involved. Rarely, interventions target training for the person with dementia directly, with the aim of assessing the extent to which this reduces the burden on carers.

**Geographical distribution.** The 48 included studies were conducted in 12 countries. Nineteen studies were conducted in China (37.3%), eight studies in Turkey, six in Iran, five in Brazil, three in India, two in Egypt and one each in Argentina, Colombia, Peru, Thailand and Vietnam. The majority of trials were conducted in regions of those countries that are more economically developed than the national average (Figure S1).

**Study designs.** Except for two three‐arm studies, all included studies were two‐arm parallel‐group RCTs [43, 44]. Most studies compared the intervention arm with no intervention, while other comparators, including educational materials, limited education, unstructured group sessions, or other interventions were also used. Only four studies used cluster randomisation [9, 45, 46, 47], while the rest were randomised at the level of individual carers or patients, or randomised at the level of carer‐care recipient dyads.

**Outcome measurement.** We identified 67 carer outcomes and 36 care recipient outcomes in the included trials, yet only 15 studies measured outcomes for *both* carers and care recipients (Table S3). Twenty‐three studies only measured carer outcomes, and 10 studies only measured care recipient outcomes. Consistent with previous studies [23, 25], perceived burden and depression were the most frequently used carer outcomes, measured in 24 and 10 studies, respectively. Care recipient outcomes focused on quality of life (QoL) and neuropsychiatric symptoms, measured in 13 and 11 studies respectively. We also assessed the appropriateness of measurement instruments. Govindakumari et al. (2020) [48] did not report the instruments used. SF36 total scores were misused as a measurement of QoL (erroneously computing a single, combined measure [49]) in three trials [50, 51, 52]. These data were excluded from meta‐analyses.

### 3.3 Intervention Characteristics

**Mode.** Most studies required face‐to‐face interaction, such as group meetings, home visits and individual sessions, except for six studies delivered remotely. Telephone interviews were the most frequently used remote delivery mode, appearing in 15 studies, but 13 studies combined this technique with face‐to‐face interaction. In 27 studies, the intervention was delivered weekly, and only one study did not deliver the intervention regularly but used a self‐paced online learning platform. Interventions typically consisted of regular group training sessions and home visits, and occasionally support groups and counselling. Outside of China, there was only one trial for a dyadic intervention (in Brazil). Four studies used instant message software, for example WeChat and WhatsApp, in the intervention. Three studies used DVDs/CDs for training purposes [45, 53, 54, 55]. Notably, Baruah et al. (2021) examined the effectiveness of iSupport, an online intervention developed by the World Health Organization (WHO), with a pilot RCT in India [56, 57, 58].

**Feasibility.** Study attrition ranged from 0% to 64.4%, with a median of 9.6%. Among 30 studies reporting on their attrition, 50% reported attrition rates > 10%. Common reasons included deaths of people with dementia, loss of contact and relocation to another city. Two studies reported a high attrition rate caused by high mortality, despite the exclusion of severe cases [9, 59], which was likely caused by the low quality of healthcare. One study associated higher attrition rates with the greater age of carers [60]. One study attributed the low attrition rate achieved to the short disease courses [61].

**Scalability.** Only seven interventions were validated across settings, including the ‘Helping Carers to Care’ intervention [59, 62, 63], the REACH intervention [9, 10, 11], the PLST‐based intervention [64, 65, 66], the mutual support group intervention [61, 67, 68], group spiritual care [43, 69, 70], and reminiscence therapy for people with dementia [44, 71, 72]. In China, dyadic interventions, rather than interventions that focused on carers alone, were the most commonly used intervention type.

### 3.4 Participant Characteristics

Most studies were conveniently sampled from hospitals or patient registries, while 12 studies recruited from the community and four recruited from care homes (Table S4). The median group size was 35. Two trials did not involve people with dementia but focused on professional carers. Among the 34 studies that provided data, the median female percentage among people with dementia was 57.6%. Among the 35 studies reporting on carers, the median female percentage was 78.7%.

### 3.5 Risk of Bias

**FIGURE 2:**
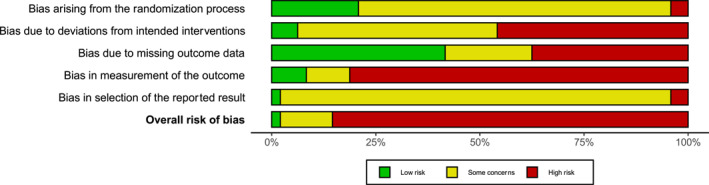
Summary of risk of bias assessments. Domain‐level results for each study are also available as a traffic light plot in Table S5.

Risk of bias of individual studies is summarised in Figure 2 and Tables S5. Only one study was judged to have a low overall risk of bias, due to its successful cluster randomisation design and blinding of both assessors and participants [9]. Most trials did not provide sufficient information to assess risk of bias for all domains. For example, only nine studies described randomisation with reasonable details required by RoB2. The participants were typically not blinded and their self‐reported outcomes may be affected by the knowledge of their assigned interventions, leading to a high risk of bias in outcome measurement, because most psychometric instruments were self‐reported [73].

The funnel plot and Egger's test showed that the data for perceived burden were subject to publication bias, while funnel plots of other outcomes (neuropsychiatric symptoms and cognitive function of people with dementia) did not have enough data to evaluate publication bias for Egger's test (Table S6 and Figure S2).

### 3.6 Pairwise Meta‐Analysis

**FIGURE 3:**
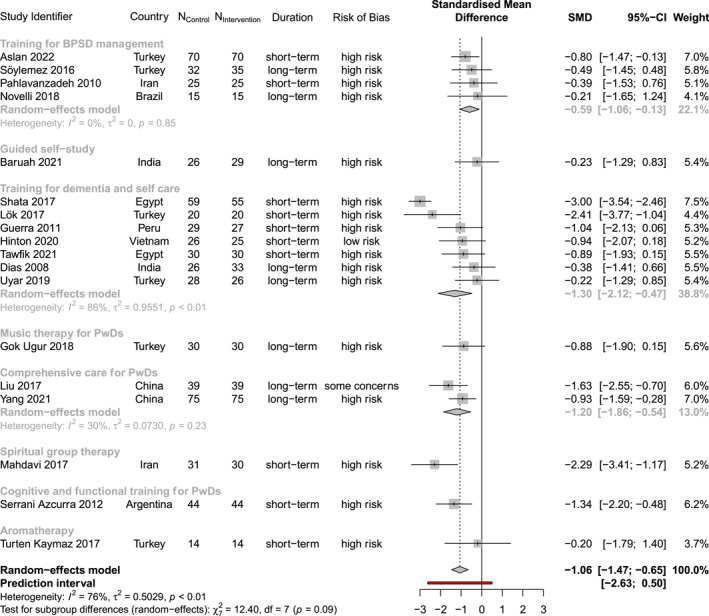
Forest plot of intervention effects on perceived burden. Both subgroup (in light grey) and combined (in black) results are visualised.

**FIGURE 4:**
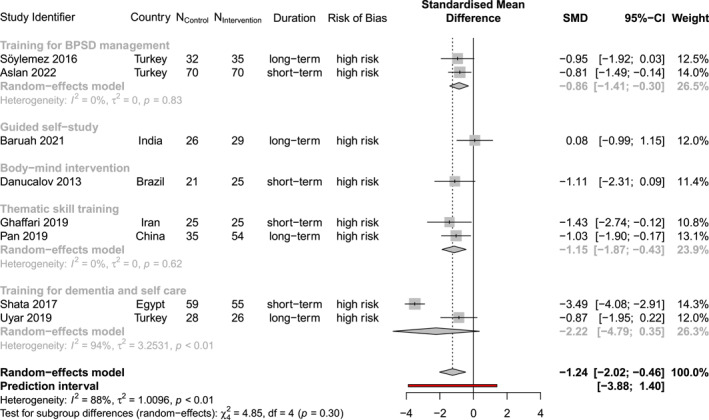
Forest plot of intervention effects on carer depression. Both subgroup (in light grey) and combined (in black) results are visualised.

Of all outcome data collected, nine outcomes were measured in at least five studies, after removing studies measuring the outcome with unclear or wrong instruments. Thus, we conducted meta‐analyses for five carer outcomes, namely perceived burden, care‐related distress, depression, QoL, and anxiety, and four care recipient outcomes, namely neuropsychiatric symptoms, ADL, cognitive function and QoL (Table 2 and Figure S3). Table 2 shows the overall results of the meta‐analysis, with individual results for primary outcomes visualised in Figures 3 and 4. Regardless of intervention content, interventions to support carers' health generally led to significant improvements in both the meta‐analysed carer and care recipient outcomes.

**TABLE 2:** Summary of meta‐analysis results after removing influential cases.

	*k*	*N*	TE	95% CI	95% PI	*I* ^2^	95% CI	Excluded study^a^
Caregiver outcomes								
Perceived Burden	17	1127	−0.9109	[−1.1697, −0.6522]	[−1.3932, −0.4287]	0.2475	[0.0000, 0.5806]	Shata 2017
Depression	7	501	−0.8472	[−1.2097, −0.4848]	[−1.3226, −0.3719]	0	[0.0000, 0.7081]	Shata 2017
Care‐related distress	8	484	−0.4433	[−0.8120, −0.0745]	[−0.9036, 0.0171]	0	[0.0000, 0.6758]	Shata 2017
Quality Of life	7	446	0.5589	[0.1027, 1.0151]	[−0.4446, 1.5625]	0.2423	[0.0000, 0.6664]	Wang 2012
Anxiety	4	220	−1.4136	[−2.1659, −0.6614]	[−4.1699, 1.3426]	0.4565	[0.0000, 0.8191]	Shata 2017
Care recipient outcomes								
Neuropsychiatric Symptoms	11	777	−0.6817	[−0.9704, −0.3930]	[−1.0149, −0.3484]	0	[0.0000, 0.6023]	None
Cognitive Function	9	782	0.7603	[0.4688, 1.0519]	[0.3836, 1.1371]	0	[0.0000, 0.6480]	JIANG 2012
Quality of life	9	686	0.6889	[0.3755, 1.0023]	[0.3107, 1.0670]	0	[0.0000, 0.6480]	None
Composite activities of daily life	5	437	−0.7967	[−1.1992, −0.3942]	[−1.4502, −0.1432]	0	[0.0000, 0.7920]	Wang 2014

^a^ The excluded studies were identified through influence diagnostics (Figure S4), which revealed that certain studies had a disproportionately large impact on the meta‐analytic results compared to others with similar stated RCT designs, as can be seen in Figures 3, 4 and S6. These studies were considered extreme cases or outliers, contributing substantially to the observed heterogeneity in intervention effects. Their outsized influence may indicate underlying, unreported yet significant differences between studies. In contrast, Figures 3, 4 and S3 present meta‐analytic and individual results without removing influential cases.

The meta‐analysis of 18 trials involving 1241 carers demonstrated a significant reduction in perceived burden with an effect estimate of −1.0619 (95% CI: [−1.4695, −0.6543], *I* ^2^: 75.59%). After removing the influential case by Shata et al. (2017) in a secondary analysis, the effect size narrowed to −0.9109 (95% CI: [−1.1697, −0.6522], *I* ^2^: 24.75%). The prediction interval (PI), initially ranging from −2.6286 to 0.5048, later narrowed to −1.3932 to − 0.4287 after removing outliers, suggesting a potential positive effect on perceived burden in future research.

Across 8 trials with 615 carers, interventions indicated a significant reduction in depression with an effect size of −1.2408 (95% CI: [−2.0180, −0.4635], *I* ^2^: 88.47%). Excluding Shata et al. (2017), the effect size decreased to −0.8472 (95% CI: [−1.2097, −0.4848], *I* ^2^: 0%). The PI spanned from −3.8840 to 1.4024 originally but tightened to a range of −1.3226 to −0.3719, which suggests the positive results could likely be replicated in future trials.

Based on 8 trials with 484 participants, the interventions had an effect size of −0.4433 in reducing care‐related distress (95% CI: [−0.8120, −0.0745], *I* ^2^: 0%). Excluding Shata et al. (2017), the effects remained consistent. The PI, from −0.9036 to 0.0171, implies that any future studies' results would likely be mixed.

Eight trials encompassing 524 carers indicated an improvement in QoL with an effect size of 0.8216 (95% CI: [0.1398, 1.5034], *I* ^2^: 70.34%). However, the robustness of this result is not assured when omitting the data of Liu et al. (2017). Excluding Wang (2012), the effect size was reduced to 0.5589 (95% CI: [0.1027, 1.0151], *I* ^2^: 24.23%). The PI went from an original range of −1.3444 to 2.9876 to a narrower −0.4446 to 1.5625, which suggests a mixed result for future studies.

For anxiety, 5 trials with 334 carers revealed a reduction in anxiety levels with an effect estimate of −1.9926 (95% CI: [−3.1975, −0.7876], *I* ^2^: 90.15%). Excluding Shata (2017), the effect size was modified to −1.4136 (95% CI: [−2.1659, −0.6614], *I* ^2^: 45.65%). The PI transitioned from −6.4781 to 2.4930, becoming −4.1699 to 1.3426 after the removal, which again indicates a more predictable and narrower range for anticipated future study results.

From 11 trials with 777 care recipients, interventions showed a significant reduction in neuropsychiatric symptoms with an effect size of −0.6817 (95% CI: [−0.9704, −0.3930], *I* ^2^: 0%). Excluding influential cases, the effect size remained consistent, indicating the robustness of the findings. The PI was originally set from −1.0149 to −0.3484, suggesting that if a new study were conducted, its results would still likely be positive.

In 10 trials with 876 participants, there was a notable improvement in cognitive function, indicated by an effect size of 0.6115 (95% CI: [0.2452, 0.9778], *I* ^2^: 39.23%). Excluding Jiang (2012), the effect size increased to 0.7603 (95% CI: [0.4688, 1.0519], *I* ^2^: 0%). The PI initially spanned from −0.3516 to 1.5746 and later adjusted to a range of 0.3836–1.1371, suggesting a reliable and consistent effectiveness in future similar studies.

Across 9 trials involving 686 care recipients, there was an observed improvement in the QoL with an effect size of 0.6889 (95% CI: [0.3755, 1.0023], *I* ^2^: 0%). No influential cases were removed for this outcome, and thus the effect remained unchanged. The PI, ranging from 0.3107 to 1.0670, indicates a consistent anticipated effect in any subsequent studies with similar interventions.

Based on 6 trials with 560 participants, there was a significant reduction in challenges related to daily activities, with an effect size of −1.0639 (95% CI: [−1.5665, −0.5614], *I* ^2^: 44.78%). Excluding Wang (2014), the effect size shifted to − 0.7967 (95% CI: [−1.1992, −0.3942], *I* ^2^: 0%). The PI started with a range of −2.4270 to 0.2991 and after exclusion narrowed to −1.4502 to −0.1432, confirming the consistency and reliability of the intervention effects in future research.

### 3.7 Exploring Heterogeneity

The variable importance plot of intervention effects on perceived burden suggests that the source of participants, the frequency of intervention, the type of diseases and treatment duration are the most important variables that contribute to heterogeneity (Figure S6). In the meta‐regression and subgroup analyses, these variables only yielded minimal impact on study heterogeneity, except for the source of participants (Figure S5 and Table S7). Participants recruited from patient organisations seem more likely to benefit from the intervention, compared to those from hospitals, communities and nursing homes.

## 4 Discussion

### 4.1 Summary Effects

This comprehensive meta‐analysis of interventions to support carers in LMICs provides evidence that interventions that provide support to carers can improve the health of both people with dementia and their carers. Despite recent increases in epidemiological and other studies of dementia in LMICs, research on interventions to support carers is still relatively rare [74], only accounting for 10% of the interventions included in recent reviews. Our review of 48 studies published between 2008 and 2022 found medium‐to‐large treatment effects on perceived burden, depression, anxiety, care‐related distress and QoL of carers. Furthermore, the interventions also led to reductions in the severity of neuropsychiatric symptoms, and improvements in ADLs, QoL and cognitive function of people with dementia, with medium‐to‐large treatment effects. The effect size was typically larger than that observed in similar studies from HICs, which could be a result of either the low quality of study designs and/or the lack of formal support for carers in the usual care comparator [15, 22]. These factors lowered the baseline for mental health status in LMICs [75]. We noted that, for perceived burden, there was a risk of publication bias, where selective reporting of positive results may exaggerate the effect size.

### 4.2 Quality of Evidence

Low‐quality study designs may have caused measurement errors and compromised randomisation and blinding, introducing biases to our results. This was consistent with our previous systematic mapping [20], as well as previous reviews that were mostly based on interventions from HICs [15, 21, 22]. Registration of trials and protocols could clearly be improved. As only one study was partially funded by a real estate developer, and there was no obvious conflict of interest observed, we believed that the risk of industry bias was low.

Despite the high risk of bias of included trials, the estimated effects are robust and may well be observed in similar studies in the future, due to high consistency across studies suggested by PIs not overlapping with zero and low heterogeneity after excluding outliers. However, caution should be exercised in generalising the results to all types of interventions as the meta‐analysis for each outcome includes different sets of interventions and each intervention is measured for different sets of outcomes. As a result, an intervention that is effective for one outcome may be ineffective for another.

### 4.3 Factors Affecting Intervention Effects

A strength of this review is that we not only assessed the quantitative evidence on treatment effects, but also considered factors that may influence the effectiveness and implementation of interventions to support carers. In the studies included in this review, carers of people with dementia were much more likely to be female (Figure S1), which aligns with the gendered role described in previous literature [5]. We also observed a higher female proportion in people with dementia, which may be explained by the slightly higher susceptibility of women to develop dementia and their greater life expectancy [76].

Other cultural aspects may also affect an intervention's effect. Notably, the lack of cultural tailoring may have resulted in the failure of the ‘Helping Carers to Care’ intervention to replicate favourable results in Peru and Russia as in India [77]. In contrast, Gok Ugur et al. (2019) [55] and Duru Aşiret et al. (2016) adapted music therapy and reminiscence therapy to Turkish culture, which improved the health of people with dementia and carers. Islamic group spiritual care in two trials [43, 69], plus the spiritual care component in an intervention for Thai Buddhists [70], will require cultural and religious adaptation.

### 4.4 Intervention Types and Targets

By qualitatively reviewing the content of the included interventions, we identified three typical therapeutic targets and five common strategies:

— Target 1: Dementia care knowledge
  • Strategy 1: Provision of knowledge and relevant information
— Target 2: Care dependency
  • Strategy 2: Preserving the cognitive function of people with dementia
  • Strategy 3: Training for behavioural and psychological symptoms in dementia (BPSD) management
— Target 3: Carers' stress‐health process
  • Strategy 4: Mind‐body interactions
  • Strategy 5: Complex skill training

First, in LMICs, carers do not typically conceptualise dementia care as a burden [35, 78]. Thus, providing basic knowledge about dementia care could raise their awareness of caregiving as a burden, encouraging them to seek help when in need. For example, Dias et al. (2008) showed that the ‘Helping Carers to Care’ intervention, which trained local informal health workers to deliver dementia care education to family carers, was effective in reducing perceived burden and improving the mental health of carers, while Guerra et al. (2011) [62] showed that the intervention improved perceived burden, but not their psychological distress or QoL in Peru. Wang et al. (2012) [61] implemented peer‐led group sessions which proved effective in reducing carer burden and improving QoL.

Second, as dementia progresses, individuals may lose their ability to self‐care, increasing their reliance on carers. Cognitive and functional training aims to slow this decline, potentially easing carers' load. Reminiscence therapy [44, 71, 72] has shown promise in improving the well‐being of those with dementia, albeit with mixed results in reducing carer burden. Managing behavioural and psychological symptoms of dementia (BPSD) adds to carer stress [7]. Dyadic interventions, such as Progressively Lowered Stress Threshold (PLST) [65, 66] and Tailored Activity Program (TAP) [64, 79, 80, 81], offer strategies to manage BPSD. Additionally, alternative therapies like music and aromatherapy have shown effectiveness in reducing BPSD and preventing increased carer burden [55, 82].

Third, interventions targeting carers' stress‐health process have gained prominence [10, 83]. Multi‐component psychoeducation programmes have emerged to equip caregivers with stress management techniques and essential skills for self‐care [84]. For example, Hinton et al. (2020) [9] found that a culturally tailored version of REACH effectively reduced carers' perceived burden, although it did not significantly impact their knowledge about dementia. Similarly, Uyar et al. (2019) [85] and Shata et al. (2017) provided dementia care knowledge and promoted carer self‐care via group meetings, which significantly reduced carers' perceived burden.

Mind‐body interventions, such as body relaxation [53, 54, 86], meditation [53, 54], mindfulness [87], and spiritual care [88, 89] were used for stress management. Norouzi et al. [87] found that mindfulness‐based cognitive therapy improved perceived burden and depression but did not enhance carers' QoL. Danucalov et al. [53, 54] demonstrated that yoga combined with compassion meditation significantly reduced stress, anxiety, depression and salivary cortisol levels while enhancing various aspects of carers' well‐being. Kamkhagi et al. [86] showed that bodily awareness therapy and psychodynamic group therapy effectively improved perceived burden, QoL and depressive symptoms, with psychodynamic group therapy more effective in reducing depression. Mahdavi et al. (2017) [43] provided spiritual care through group sessions, resulting in improved perceived burden and self‐efficacy among carers. Pankong et al. [70] integrated spiritual care into structured group training, enhancing carers' subjective well‐being, though without significant improvements in positive aspects of well‐being.

While consistent with previous literature in HICs [13, 15], multi‐component interventions typically show the most significant health improvements. Our analysis showed that comprehensive care interventions [7, 47, 50, 51, 52, 90, 91, 92, 93, 94], a form of dyadic intervention monitored and delivered by community nurses, were consistently effective, yet the interventions were resource‐intensive.

### 4.5 Limitations

Our study is subject to several limitations. Firstly, the searches were conducted 3 years prior to publication. However, the calculated prediction intervals indicate that additional studies are unlikely to change the findings. Thus, the conclusions remain robust and valid despite the emergence of newer studies.

Secondly, although we included widely indexed databases like Medline, Embase, and PsycINFO, where high‐quality research is typically published, we excluded certain regional databases, such as Latin American and Caribbean Health Sciences Literature (LILACS), limiting inclusion of research from these regions. Thus, the samples analysed may not accurately represent the general populations in LMICs. Notably, included trials were concentrated in only 12 countries, none of which were classified as low‐income countries.

Furthermore, the scalability of the interventions examined required further trials. Only three interventions were validated across different settings: the 10/66 intervention in two studies [59, 62], the REACH intervention [9], which had prior trials in the US [10], and the mutual support group [61], which had previous trials in Hong Kong [67, 68]. As most studies employed convenience sampling methods, the generalisability of the results to broader dementia patient populations may be subject to potential selection bias due to inequities in accessing care [95].

### 4.6 Future Directions

There are key avenues for enhancing the rigour and relevance of future research efforts. Firstly, acquiring comprehensive patient‐level data and addressing missing data are essential to mitigate biases and enable more robust meta‐analyses [96, 97]. Patient‐level data offer more accurate estimates of between‐group standardized mean differences, reducing the risk of overestimating intervention effectiveness. Discrepancies, like those observed with the Helping Carers to Care intervention, underscore the need for a thorough investigation into missing or incomplete datasets to better understand the efficacy of various types of carer support programmes.

Secondly, our subgroup and MetaForest‐based analysis (Table S7 and Figure S6) highlights patient sources as a major source of heterogeneity. Tailoring interventions to specific subgroups is crucial, suggesting the potential benefits of modular designs that allow for flexible combinations of interventions to meet diverse needs. However, understanding the costs associated with modular approaches remains a critical gap, particularly in resource‐limited settings. Further research needs to be done to increase trial representativeness and improve the accessibility and affordability of interventions for carers across different settings.

## 5 Conclusions

In summary, our systematic review of interventions to support carers of people with dementia in LMICs reveals a concentration of evidence generation in specific countries, a limited range of interventions studied, and a notable susceptibility to bias across the body of evidence. Given the anticipated demographic shifts in LMICs, there is a clear imperative for increased and improved research efforts to inform policies that support the crucial roles of family and other carers of people with dementia. A coordinated approach to evidence generation, both within individual countries and across borders, is essential to foster the development of robust, relevant, and impactful interventions tailored to the unique challenges faced by these regions.

## Conflicts of Interest

The STRiDE consortium was supported by UK Research and Innovation Global Challenges Research Fund (ES/P010938/1). Meizhi Li was supported by the fund from the Second Xiangya Hospital of Central South University. The sponsors did not participate in the research design, data analysis, interpretation of results, preparation of the manuscript, or the decision to submit the article for publication.

## Supporting information

Supporting Information S1

## Data Availability

Data are available from the supplementary materials. Examples of code and data collection forms and extracted data can be found at https://github.com/ydchen17/meta4carers2care.
