# Supplementary material for: Effectiveness of Interventions to Support Carers of People With Dementia in Low‐ and Middle‐Income Countries: A Systematic Review and Meta‐Analysis
Source: Int J Geriatr Psychiatry. 2025 Feb 27;40(3):e70054. doi: 10.1002/gps.70054 (PMC11867927; doi:10.1002/gps.70054)
Supplement: Supplementary file 1 — Supporting Information S1 [file GPS-40-e70054-s001.docx]

**Supplemental materials**

**Effectiveness of interventions to support carers of people with dementia in low- and middle-income countries: a systematic review and meta-analysis**

**November 2024**

Table of Contents

[Table S1. PRISMA Checklist 3](#_Toc190688781)

[Table S2. Search Strategies 6](#_Toc190688782)

[Figure S1. Characteristics of included trials 14](#_Toc190688783)

[Figure S1a. Trends of dementia caregiver intervention trials in LMICs 14](#_Toc190688784)

[Figure S1b. Geographic distribution of included trials 15](#_Toc190688785)

[Figure S1c. Human development index (HDI) of trial locations in comparison with national HDI 15](#_Toc190688786)

[Figure S1d. Mean ages and female percentages of study cohorts 16](#_Toc190688787)

[Table S3. List of most measured outcomes and measurement instruments 17](#_Toc190688788)

[Table S4. Selected characteristics of participants in included trials 18](#_Toc190688789)

[Table S5. Risk of bias assessments of individual studies 22](#_Toc190688790)

[Table S6. Egger’s test results 23](#_Toc190688791)

[Figure S2. Funnel plots 24](#_Toc190688792)

[Figure S2a. Perceived burden of carers 24](#_Toc190688793)

[Figure S2b. Neuropsychiatric symptoms of care recipients 25](#_Toc190688794)

[Figure S2c. Cognitive function of care recipients 26](#_Toc190688795)

[Figure S3. Additional forest plots 27](#_Toc190688796)

[Figure S3a. Care-related distress 27](#_Toc190688797)

[Figure S3b. Carer quality of life 27](#_Toc190688798)

[Figure S3c. Carer anxiety 28](#_Toc190688799)

[Figure S3d. Neuropsychiatric symptoms of care recipients 28](#_Toc190688800)

[Figure S3e. Cognitive function of care recipients 29](#_Toc190688801)

[Figure S3f. Quality of life of care recipients 29](#_Toc190688802)

[Figure S3g. Composite activities of daily life of care recipients 30](#_Toc190688803)

[Figure S4. Leave-one-out analysis 31](#_Toc190688804)

[Figure S4a. Care-related distress 31](#_Toc190688805)

[Figure S4b. Perceived burden of carers 31](#_Toc190688806)

[Figure S4c. Carer depression 31](#_Toc190688807)

[Figure S4d. Carers’ quality of life 32](#_Toc190688808)

[Figure S4e. Carer anxiety 32](#_Toc190688809)

[Figure S4f. Cognitive function of care recipients 32](#_Toc190688810)

[Figure S4g. Neuropsychiatric symptoms of care recipients 32](#_Toc190688811)

[Figure S4h. Quality of life of care recipients 33](#_Toc190688812)

[Figure S4i. Composite activities of daily life of care recipients 33](#_Toc190688813)

[Table S7. Summary of subgroup analyses for pre-determined factors 34](#_Toc190688814)

[Table S8. Summary of meta-regression for pre-determined factors 35](#_Toc190688815)

[Figure S5. Bubble plots of meta-regression for caregiver burden 36](#_Toc190688816)

[Figure S6. MetaForest results 37](#_Toc190688817)

[Figure S6a. Variable importance plot 37](#_Toc190688818)

[Figure S6b. Partial dependency plot 38](#_Toc190688819)

[Table S9. Citations of included studies 39](#_Toc190688820)

[Table S10. List of collaborators in the STRiDE Evidence Review Group 43](#_Toc190688821)

1. PRISMA Checklist

| Section and Topic | Item # | Checklist item | Location where item is reported |
| --- | --- | --- | --- |
| TITLE | | |  |
| Title | 1 | Identify the report as a systematic review. | Cover Page |
| ABSTRACT | | |  |
| Abstract | 2 | See the PRISMA 2020 for Abstracts checklist. | Abstract |
| INTRODUCTION | | |  |
| Rationale | 3 | Describe the rationale for the review in the context of existing knowledge. | Section 1 |
| Objectives | 4 | Provide an explicit statement of the objective(s) or question(s) the review addresses. | Section 1 |
| METHODS | | |  |
| Eligibility criteria | 5 | Specify the inclusion and exclusion criteria for the review and how studies were grouped for the syntheses. | Section 2.1 |
| Information sources | 6 | Specify all databases, registers, websites, organisations, reference lists and other sources searched or consulted to identify studies. Specify the date when each source was last searched or consulted. | Section 2.1 |
| Search strategy | 7 | Present the full search strategies for all databases, registers and websites, including any filters and limits used. | Section 2.1  Table S2 |
| Selection process | 8 | Specify the methods used to decide whether a study met the inclusion criteria of the review, including how many reviewers screened each record and each report retrieved, whether they worked independently, and if applicable, details of automation tools used in the process. | Section 2.1  Figure 1 |
| Data collection process | 9 | Specify the methods used to collect data from reports, including how many reviewers collected data from each report, whether they worked independently, any processes for obtaining or confirming data from study investigators, and if applicable, details of automation tools used in the process. | Section 2.1  Section 2.3 |
| Data items | 10a | List and define all outcomes for which data were sought. Specify whether all results that were compatible with each outcome domain in each study were sought (e.g. for all measures, time points, analyses), and if not, the methods used to decide which results to collect. | Section 2.1 |
|  | 10b | List and define all other variables for which data were sought (e.g. participant and intervention characteristics, funding sources). Describe any assumptions made about any missing or unclear information. | Section 2.1 |
| Study risk of bias assessment | 11 | Specify the methods used to assess risk of bias in the included studies, including details of the tool(s) used, how many reviewers assessed each study and whether they worked independently, and if applicable, details of automation tools used in the process. | Section 2.2 |
| Effect measures | 12 | Specify for each outcome the effect measure(s) (e.g. risk ratio, mean difference) used in the synthesis or presentation of results. | Section 2.3 |
| Synthesis methods | 13a | Describe the processes used to decide which studies were eligible for each synthesis (e.g. tabulating the study intervention characteristics and comparing against the planned groups for each synthesis (item #5)). | Section 2.4  Section 2.5 |
|  | 13b | Describe any methods required to prepare the data for presentation or synthesis, such as handling of missing summary statistics, or data conversions. | Section 2.4  Section 2.5 |
|  | 13c | Describe any methods used to tabulate or visually display results of individual studies and syntheses. | Section 2.4  Section 2.5 |
|  | 13d | Describe any methods used to synthesize results and provide a rationale for the choice(s). If meta-analysis was performed, describe the model(s), method(s) to identify the presence and extent of statistical heterogeneity, and software package(s) used. | Section 2.5 |
|  | 13e | Describe any methods used to explore possible causes of heterogeneity among study results (e.g. subgroup analysis, meta-regression). | Section 2.5 |
|  | 13f | Describe any sensitivity analyses conducted to assess robustness of the synthesized results. | Section 2.5 |
| Reporting bias assessment | 14 | Describe any methods used to assess risk of bias due to missing results in a synthesis (arising from reporting biases). | Section 2.2 |
| Certainty assessment | 15 | Describe any methods used to assess certainty (or confidence) in the body of evidence for an outcome. | Section 2.2  Section 2.5 |
| RESULTS | | |  |
| Study selection | 16a | Describe the results of the search and selection process, from the number of records identified in the search to the number of studies included in the review, ideally using a flow diagram. | Section 3.1  Figure 1 |
|  | 16b | Cite studies that might appear to meet the inclusion criteria, but which were excluded, and explain why they were excluded. | Table S9 |
| Study characteristics | 17 | Cite each included study and present its characteristics. | Table 1  Table S7 |
| Risk of bias in studies | 18 | Present assessments of risk of bias for each included study. | Table S5 (also in Figures 3, 4, S3) |
| Results of individual studies | 19 | For all outcomes, present, for each study: (a) summary statistics for each group (where appropriate) and (b) an effect estimate and its precision (e.g. confidence/credible interval), ideally using structured tables or plots. | Figures 3, 4, S3 |
| Results of syntheses | 20a | For each synthesis, briefly summarise the characteristics and risk of bias among contributing studies. | Figures 2, 3, 4, S3  Section 3.6 |
|  | 20b | Present results of all statistical syntheses conducted. If meta-analysis was done, present for each the summary estimate and its precision (e.g. confidence/credible interval) and measures of statistical heterogeneity. If comparing groups, describe the direction of the effect. | Figures 3, 4, S3  Section 3.6 |
|  | 20c | Present results of all investigations of possible causes of heterogeneity among study results. | Figure S4-6  Table S6  Section 3.7 |
|  | 20d | Present results of all sensitivity analyses conducted to assess the robustness of the synthesized results. | Figures S4, S5, S6  Tables S7, S8 |
| Reporting biases | 21 | Present assessments of risk of bias due to missing results (arising from reporting biases) for each synthesis assessed. | Table S6  Figure S2 |
| Certainty of evidence | 22 | Present assessments of certainty (or confidence) in the body of evidence for each outcome assessed. | Sections 3.5, 4.2  Figures 3, 4, S3 |
| DISCUSSION | | |  |
| Discussion | 23a | Provide a general interpretation of the results in the context of other evidence. | Sections 4.1, 4.3 |
|  | 23b | Discuss any limitations of the evidence included in the review. | Section 4.2  Section 4.5 |
|  | 23c | Discuss any limitations of the review processes used. | Section 4.5 |
|  | 23d | Discuss implications of the results for practice, policy, and future research. | Section 4.6  Section 5 |
| OTHER INFORMATION | | |  |
| Registration and protocol | 24a | Provide registration information for the review, including register name and registration number, or state that the review was not registered. | Abstract |
|  | 24b | Indicate where the review protocol can be accessed, or state that a protocol was not prepared. | Section 2.1 |
|  | 24c | Describe and explain any amendments to information provided at registration or in the protocol. | Section 2.1 |
| Support | 25 | Describe sources of financial or non-financial support for the review, and the role of the funders or sponsors in the review. | Acknowledgements |
| Competing interests | 26 | Declare any competing interests of review authors. | Acknowledgements |
| Availability of data, code and other materials | 27 | Report which of the following are publicly available and where they can be found: template data collection forms; data extracted from included studies; data used for all analyses; analytic code; any other materials used in the review. | See Notes below |

Notes

- The checklist above is take from: Page MJ, McKenzie JE, Bossuyt PM, et al. The Prisma 2020 statement: An updated guideline for reporting systematic reviews. *BMJ*. Published online March 29, 2021. doi:10.1136/bmj.n71
- Data are available from the citations listed in Table S7. Examples of code and data collection forms and extracted data can be found at <https://github.com/ydchen17/meta4carers2care>.

1. Search Strategies

| Ovid MEDLINE(R) ALL <1946 to September 20, 2022> | |
| --- | --- |
| Condition | |
| 1 | exp Dementia/ or Pick Disease of the Brain/ or Huntington Disease/ or Lewy Body Disease/ or Cognitive Dysfunction/ or (dement* or amentia* or (major adj3 cognit* adj3 disorder*) or alzheimer* or alzeimer* or (cortical adj4 sclerosis) or ((encephalopath* or cogniti* or neurocogniti*) adj4 (aids or acquired immun?deficiency syndrome* or acquired immun? deficiency syndrome* or hiv or human immun?deficiency virus* or human immun? deficiency virus*)) or pick* disease* or (lobar adj3 atroph* adj3 brain) or Huntington* disease* or Huntington* chorea or (Lewy bod* adj3 disease*) or (cerebr* adj3 deteriorat*) or (cerebr* adj3 insufficien*) or ((frontotemporal or fronto temporal or corticobasal or cortico basal or frontal lobe*) adj4 (degenerati* or dysfunction*)) or ((cognit* or memory or cerebr*) adj3 (declin* or impair* or los* or deteriorat* or degenerat* or insufficen*)) or MCI or (mild adj2 cognit* impair*)).ti,ab,kf,kw. |
| 2 | exp Cognitive Therapy/ or exp Drug Therapy/ or exp Cholinesterase Inhibitors/ or exp Antipsychotic Agents/ or exp Serotonin Uptake Inhibitors/ or exp Benzodiazepines/ or exp "Hypnotics and Sedatives"/ or exp Exercise/ or exp Exercise Therapy/ or exp Psychotherapy/ or exp Counseling/ or Psychosocial Support Systems/ or exp Complementary Therapies/ or Phototherapy/ or exp Advance Care Planning/ or Case Management/ or Caregivers/ed, px or Self-Help Groups/ or exp Social Support/ or Diagnosis, Computer-Assisted/ or Telemedicine/ or exp Computers, Handheld/ or (intervention* or therap* or treatment* or program* or manage* or prevent* or diagnos* or polic* or (cognit* adj3 (therap* or training or rehab*)) or cognit* psycho therap* or cognit* psychotherap* or drug* or medicine* or pharmacotherap* or pharmaco* therap* or cholinesterase agent* or cholinesterase inhibitor* or (tranquili* adj3 (agent* or drug*)) or antipsychotic* or (neuroleptic adj3 (agent* or drug*)) or serotonin uptake inhibitor* or serotonin reuptake inhibitor* or ssri* or benzodiazepine* or (sedative adj3 (effect* or agent*)) or memantine or donepezil or rivastigmine or galantamine or souvenaid or risperidone or haloperidol or olanzapine or quetiapine or citalopram or dextromethorphan or carbamazepine or mirtazapine or sertraline or moclobemide or trazodone or melatonin or ramelteon or methylphenidate or exercis* or physical activit* or physical training or aerobic* or arobic* or kinesi?therap* or kinesi? therap* or (social adj3 (activit* or engag* or stimul*)) or psycholog* therap* or psychotherap* or ((behavio?r* or conditioning) adj3 therap*) or counsel?ing or ((psychosocial or psycho social) adj3 (support or interven* or care)) or ((alternative or compl?ment* or traditional) adj3 (medicine* or therap*)) or acupunct* or (herb* adj3 (tea* or remedy or remedies or medicine*)) or gingko or homeopath* or ((music or art or aroma or light or photo or pet or pets) adj3 therap*) or aromatherap* or phototherap* or massage* or (mind adj3 body) or (advance? adj3 (care or medical or healthcare) adj3 plan*) or (decision* adj3 (aid* or support)) or (communicati* adj3 skill* adj3 training) or (dementia care adj3 map*) or ((person* or patient*) adj3 cent* adj3 care) or "Resources for Enhancing Alzheimer’s Caregiver Health" or "Strategies for Relatives" or ((caregiver* or carer*) adj3 educat*) or telemedicine or tele medicine or (smart adj2 (phone* or device* or tablet*)) or smartphone* or cognitive aid* or reminder* or robot*).ti,ab,kf,kw. |
| 3 | (Randomized Controlled Trial or Controlled Clinical Trial).pt. or (randomi#ed or placebo or randomly or trial or groups).ti,ab. or drug therapy.fs. |
| 4 | Developing Countries.sh,kf,kw. or (Africa* or Asia* or Caribbean* or West Indies or South America* or Latin America* or Central America* or Afghan* or Albania* or Algeria* or Angola* or Anguilla* or Antigua* or Barbuda* or Argentin* or Armenia* or Azerbaijan* or Azeri or Bangladesh* or Barbad* or Benin* or Byelarus or Byelorussian or Belarus* or Belorussia* or Belize* or Bhutan* or Bolivia* or Bosnia* or Herzegovin* or Hercegovin* or Botswana or Botsuana or Motswana or Batswana or Brasil* or Brazil* or Burkina Faso or Burkina Fasso or Burkina* or Burundi* or Urundi* or Cambodia* or Cameroon* or Cameron* or Cape Verd* or Cabo Verde or Central African Republic or Chad* or Tchad* or Chile* or China or Chinese or Colombia* or Columbia* or Comoros or Comoro Islands or Comores or Comoran or Mayotte or Congo* or Costa Rica* or Cote d'Ivoire or Ivory Coast or Ivorian* or Cook Islands or Cuba* or Croat* or Djibouti* or Dominica* or East Timor or East Timur or Timor Leste or Timorese or Ecuador* or Equador* or Egypt* or El Salvador or Salvadoran or Eritrea* or Ethiopia* or Fiji* or Gabon* or Gambia* or Gaza or Georgia Republic or Georgian or Abkhazia* or Abchasia* or South Ossetia* or Ghana* or Grenada or Grenadian or Guatemala* or Guinea* or Guinea Bissau or Guian* or Guyana or Haiti* or Hondura* or India or Indian or Indonesia* or Iran* or Iraq* or Jamaica* or Jordan* or Kazakhstan* or Kazakh or Kenya* or Kiribati or Korea* or Kosovo or Kosova* or Kyrgyzstan or Kirghizia or Kyrgyz or Kirghiz or Kirgizstan or Lao PDR or Laos or Laotian or Lebanon or Lebanese or Lesotho or Mosotho or Basotho or Liberia* or Libya* or Macedonia* or FYROM or Madagasca* or Malagasy or Malaysia* or Malaya* or Malay or Sabah or Sarawak or Malawi* or Maldives or Maldivan or Mali or Malian or Marshall Islands or Marshallese or Mauritania* or Mauriti* or Agalega Islands or Mexico or Mexican or Micronesia* or Middle East* or Moldova* or Moldovia* or Transnistria* or Mongolia* or Montenegr* or Montserrat* or Morocc* or Mozambique or Mozambican or Myanmar* or Myanma or Burma or Burmese or Namibia* or Nauru* or Niue or Nepal* or Nicaragua* or Niger or Nigerien or Nigeria* or Oman* or Pakistan* or Palau* or Palestine or Palestinian or Panama or Panamanian or Paraguay* or Papua New Guinea* or Peru or Peruvian or Philippines or Philipines or Phillipines or Phillippines or Filipino or Philipino or Philippino or Phillipino or Phillippino or Rwanda* or Ruanda* or Saint Helen* or St Helen* or Saint Kitts or St Kitts or Kittian or Nevis* or Saint Lucia* or St Lucia* or Saint Vincent or St Vincent or Vicentian or Grenadines or Samoa* or Sao Tome* or Senegal* or Serbia* or Seychell* or Sierra Leone* or Sri Lanka* or Ceylon or Solomon Island* or Somali* or South Africa* or Sudan or Sudanese or Surinam* or Swaziland or Swazi or Eswatini or Syria or Syrian or Tajikistan or Tadzhikistan or Tadjikistan or Tajik or Tadzhik or Tanzania* or Thailand or Thai or Togo or Togolese or Tonga* or Tunisia* or Tokelau or Trinidad* or Tobago* or Turkey or Turkish or Turks or Turkmenistan or Turkmen or Tuvalu* or Uganda* or Ukraine or Ukrainian or Uruguay* or Uzbekistan* or Uzbek or Vanuatu or Venezuela* or Vietnam* or Viet Nam or Wallis Futuna or West Bank or Yemen* or Zambia* or Zimbabwe*).hw,kf,kw,ti,ab,cp. or (((developing or less* developed or under developed or underdeveloped or middle income or low* income or underserved or under served or deprived or poor*) adj (countr* or nation? or population? or world)) or ((developing or less* developed or under developed or underdeveloped or middle income or low* income) adj (economy or economies)) or (low* adj (gdp or gnp or gross domestic or gross national)) or (low adj3 middle adj3 countr*) or lmic or lmics or third world or lami countr* or transitional countr*).ti,ab. |
| 5 | 1 and 2 and 3 and 4 |
| 6 | exp Animals/ not Humans/ |
| 7 | 5 not 6 |
| 8 | (Comment or Editorial or Letter).pt. |
| 9 | 7 not 8 |
| 10 | limit 9 to yr="2019 - 2020" |
| Embase <1974 to 2022 September 20> | |
| Condition | |
| 1 | exp Dementia/ or Cognitive defect/ or (dement* or amentia* or (major adj3 cognit* adj3 disorder*) or alzheimer* or alzeimer* or (cortical adj4 sclerosis) or ((encephalopath* or cogniti* or neurocogniti*) adj4 (aids or acquired immun?deficiency syndrome* or acquired immun? deficiency syndrome* or hiv or human immun?deficiency virus* or human immun? deficiency virus*)) or pick* disease* or (lobar adj3 atroph* adj3 brain) or Huntington* disease* or Huntington* chorea or (Lewy bod* adj3 disease*) or (cerebr* adj3 deteriorat*) or (cerebr* adj3 insufficien*) or ((frontotemporal or fronto temporal or corticobasal or cortico basal or frontal lobe*) adj4 (degenerati* or dysfunction*)) or ((cognit* or memory or cerebr*) adj3 (declin* or impair* or los* or deteriorat* or degenerat* or insufficen*)) or MCI or (mild adj2 cognit* impair*)).ti,ab,kf,kw. |
| 2 | exp Cognitive Therapy/ or exp Drug Therapy/ or exp Cholinesterase Inhibitors/ or exp Neuroleptic Agent/ or exp Serotonin Uptake Inhibitor/ or exp Benzodiazepine Derivative/ or Sedative Agent/ or exp Exercise/ or exp Kinesiotherapy/ or exp Psychotherapy/ or exp Counseling/ or Psychosocial Care/ or exp Alternative Medicine/ or Phototherapy/ or Advance Care Planning/ or Living Will/ or Case Management/ or Caregiver Support/ or Self Help/ or exp Social Support/ or Computer Assisted Diagnosis/ or Telemedicine/ or Telemonitoring/ or Telerehabilitation/ or Telediagnosis/ or Personal Digital Assistant/ or Smartphone/ or (intervention* or therap* or treatment* or program* or manage* or prevent* or diagnos* or polic* or (cognit* adj3 (therap* or training or rehab*)) or cognit* psycho therap* or cognit* psychotherap* or drug* or medicine* or pharmacotherap* or pharmaco* therap* or cholinesterase agent* or cholinesterase inhibitor* or (tranquili* adj3 (agent* or drug*)) or antipsychotic* or (neuroleptic adj3 (agent* or drug*)) or serotonin uptake inhibitor* or serotonin reuptake inhibitor* or ssri* or benzodiazepine* or (sedative adj3 (effect* or agent*)) or memantine or donepezil or rivastigmine or galantamine or souvenaid or risperidone or haloperidol or olanzapine or quetiapine or citalopram or dextromethorphan or carbamazepine or mirtazapine or sertraline or moclobemide or trazodone or melatonin or ramelteon or methylphenidate or exercis* or physical activit* or physical training or aerobic* or arobic* or kinesi?therap* or kinesi? therap* or (social adj3 (activit* or engag* or stimul*)) or psycholog* therap* or psychotherap* or ((behavio?r* or conditioning) adj3 therap*) or counsel?ing or ((psychosocial or psycho social) adj3 (support or interven* or care)) or ((alternative or compl?ment* or traditional) adj3 (medicine* or therap*)) or acupunct* or (herb* adj3 (tea* or remedy or remedies or medicine*)) or gingko or homeopath* or ((music or art or aroma or light or photo or pet or pets) adj3 therap*) or aromatherap* or phototherap* or massage* or (mind adj3 body) or (advance? adj3 (care or medical or healthcare) adj3 plan*) or (decision* adj3 (aid* or support)) or (communicati* adj3 skill* adj3 training) or (dementia care adj3 map*) or ((person* or patient*) adj3 cent* adj3 care) or "Resources for Enhancing Alzheimer’s Caregiver Health" or "Strategies for Relatives" or ((caregiver* or carer*) adj3 educat*) or telemedicine or tele medicine or (smart adj2 (phone* or device* or tablet*)) or smartphone* or cognitive aid* or reminder* or robot*).ti,ab,kf,kw. |
| 3 | (Randomized Controlled Trial/ or Controlled Clinical Trial/ or random*.ti,ab. or Randomization/ or Intermethod Comparison/ or placebo.ti,ab. or (compare or compared or comparison).ti. or ((evaluated or evaluate or evaluating or assessed or assess) and (compare or compared or comparing or comparison)).ab. or (open adj label).ti,ab. or ((double or single or doubly or singly) adj (blind or blinded or blindly)).ti,ab. or Double Blind Procedure/ or parallel group*1.ti,ab. or (crossover or cross over).ti,ab. or ((assign* or match or matched or allocation) adj5 (alternate or group*1 or intervention*1 or patient*1 or subject*1 or participant*1)).ti,ab. or (assigned or allocated).ti,ab. or (controlled adj7 (study or design or trial)).ti,ab. or (volunteer or volunteers).ti,ab. or Human Experiment/ or trial.ti.) not (((random* adj sampl* adj7 (cross section* or questionnaire*1 or survey* or database*1)).ti,ab. not (Comparative Study/ or Controlled Study/ or randomi?ed controlled.ti,ab. or randomly assigned.ti,ab.)) or (Cross-Sectional Study/ not (Randomized Controlled Trial/ or Controlled Clinical Study/ or Controlled Study/ or randomi?ed controlled.ti,ab. or control group*1.ti,ab.)) or (((case adj control*) and random*) not randomi?ed controlled).ti,ab. or (systematic review not (trial or study)).ti. or (nonrandom* not random*).ti,ab. or random field*.ti,ab. or (random cluster adj3 sampl*).ti,ab. or ((review.ab. and review.pt.) not trial.ti.) or (we searched.ab. and (review.ti. or review.pt.)) or update review.ab. or (databases adj4 searched).ab. or ((rat or rats or mouse or mice or swine or porcine or murine or sheep or lambs or pigs or piglets or rabbit or rabbits or cat or cats or dog or dogs or cattle or bovine or monkey or monkeys or trout or marmoset*1).ti. and Animal Experiment/) or (Animal Experiment/ not (Human Experiment/ or Human/))) |
| 4 | Developing Countries.sh,kf,kw. or (Africa* or Asia* or Caribbean* or West Indies or South America* or Latin America* or Central America* or Afghan* or Albania* or Algeria* or Angola* or Anguilla* or Antigua* or Barbuda* or Argentin* or Armenia* or Azerbaijan* or Azeri or Bangladesh* or Barbad* or Benin* or Byelarus or Byelorussian or Belarus* or Belorussia* or Belize* or Bhutan* or Bolivia* or Bosnia* or Herzegovin* or Hercegovin* or Botswana or Botsuana or Motswana or Batswana or Brasil* or Brazil* or Burkina Faso or Burkina Fasso or Burkina* or Burundi* or Urundi* or Cambodia* or Cameroon* or Cameron* or Cape Verd* or Cabo Verde or Central African Republic or Chad* or Tchad* or Chile* or China or Chinese or Colombia* or Columbia* or Comoros or Comoro Islands or Comores or Comoran or Mayotte or Congo* or Costa Rica* or Cote d'Ivoire or Ivory Coast or Ivorian* or Cook Islands or Cuba* or Croat* or Djibouti* or Dominica* or East Timor or East Timur or Timor Leste or Timorese or Ecuador* or Equador* or Egypt* or El Salvador or Salvadoran or Eritrea* or Ethiopia* or Fiji* or Gabon* or Gambia* or Gaza or Georgia Republic or Georgian or Abkhazia* or Abchasia* or South Ossetia* or Ghana* or Grenada or Grenadian or Guatemala* or Guinea* or Guinea Bissau or Guian* or Guyana or Haiti* or Hondura* or India or Indian or Indonesia* or Iran* or Iraq* or Jamaica* or Jordan* or Kazakhstan* or Kazakh or Kenya* or Kiribati or Korea* or Kosovo or Kosova* or Kyrgyzstan or Kirghizia or Kyrgyz or Kirghiz or Kirgizstan or Lao PDR or Laos or Laotian or Lebanon or Lebanese or Lesotho or Mosotho or Basotho or Liberia* or Libya* or Macedonia* or FYROM or Madagasca* or Malagasy or Malaysia* or Malaya* or Malay or Sabah or Sarawak or Malawi* or Maldives or Maldivan or Mali or Malian or Marshall Islands or Marshallese or Mauritania* or Mauriti* or Agalega Islands or Mexico or Mexican or Micronesia* or Middle East* or Moldova* or Moldovia* or Transnistria* or Mongolia* or Montenegr* or Montserrat* or Morocc* or Mozambique or Mozambican or Myanmar* or Myanma or Burma or Burmese or Namibia* or Nauru* or Niue or Nepal* or Nicaragua* or Niger or Nigerien or Nigeria* or Oman* or Pakistan* or Palau* or Palestine or Palestinian or Panama or Panamanian or Paraguay* or Papua New Guinea* or Peru or Peruvian or Philippines or Philipines or Phillipines or Phillippines or Filipino or Philipino or Philippino or Phillipino or Phillippino or Rwanda* or Ruanda* or Saint Helen* or St Helen* or Saint Kitts or St Kitts or Kittian or Nevis* or Saint Lucia* or St Lucia* or Saint Vincent or St Vincent or Vicentian or Grenadines or Samoa* or Sao Tome* or Senegal* or Serbia* or Seychell* or Sierra Leone* or Sri Lanka* or Ceylon or Solomon Island* or Somali* or South Africa* or Sudan or Sudanese or Surinam* or Swaziland or Swazi or Eswatini or Syria or Syrian or Tajikistan or Tadzhikistan or Tadjikistan or Tajik or Tadzhik or Tanzania* or Thailand or Thai or Togo or Togolese or Tonga* or Tunisia* or Tokelau or Trinidad* or Tobago* or Turkey or Turkish or Turks or Turkmenistan or Turkmen or Tuvalu* or Uganda* or Ukraine or Ukrainian or Uruguay* or Uzbekistan* or Uzbek or Vanuatu or Venezuela* or Vietnam* or Viet Nam or Wallis Futuna or West Bank or Yemen* or Zambia* or Zimbabwe*).hw,kf,kw,ti,ab,cp. or (((developing or less* developed or under developed or underdeveloped or middle income or low* income or underserved or under served or deprived or poor*) adj (countr* or nation? or population? or world)) or ((developing or less* developed or under developed or underdeveloped or middle income or low* income) adj (economy or economies)) or (low* adj (gdp or gnp or gross domestic or gross national)) or (low adj3 middle adj3 countr*) or lmic or lmics or third world or lami countr* or transitional countr*).ti,ab. |
| 5 | 1 and 2 and 3 and 4 |
| 6 | exp Animal/ or exp Animal Experiment/ or Nonhuman/ |
| 7 | exp Human/ or exp Human Experiment/ |
| 8 | 6 not 7 |
| 9 | 5 not 8 |
| 10 | (Editorial or Letter).pt. |
| 11 | 9 not 10 |
| 12 | limit 11 to yr="2019 - 2020" |
| Global Health <1973 to 2022 Week 37> | |
| Condition | |
| 1 | exp Dementia/ or (dement* or amentia* or (major adj3 cognit* adj3 disorder*) or alzheimer* or alzeimer* or (cortical adj4 sclerosis) or ((encephalopath* or cogniti* or neurocogniti*) adj4 (aids or acquired immun?deficiency syndrome* or acquired immun? deficiency syndrome* or hiv or human immun?deficiency virus* or human immun? deficiency virus*)) or pick* disease* or (lobar adj3 atroph* adj3 brain) or Huntington* disease* or Huntington* chorea or (Lewy bod* adj3 disease*) or (cerebr* adj3 deteriorat*) or (cerebr* adj3 insufficien*) or ((frontotemporal or fronto temporal or corticobasal or cortico basal or frontal lobe*) adj4 (degenerati* or dysfunction*)) or ((cognit* or memory or cerebr*) adj3 (declin* or impair* or los* or deteriorat* or degenerat* or insufficen*)) or MCI or (mild adj2 cognit* impair*)).ti,ab. |
| 2 | exp Drug Therapy/ or exp Cholinesterase Inhibitors/ or exp Neuroleptics/ or exp Selective Serotonin Reuptake Inhibitors/ or exp Benzodiazepines/ or exp Sedatives/ or Exercise/ or Physical Therapy/ or exp Psychotherapy/ or exp Counselling/ or "Complementary and Alternative Medicine"/ or Phototherapy/ or Self Help/ or Self Care/ or exp E-health/ or Mobile Telephones/ or (intervention* or therap* or treatment* or program* or manage* or prevent* or diagnos* or polic* or (cognit* adj3 (therap* or training or rehab*)) or cognit* psycho therap* or cognit* psychotherap* or drug* or medicine* or pharmacotherap* or pharmaco* therap* or cholinesterase agent* or cholinesterase inhibitor* or (tranquili* adj3 (agent* or drug*)) or antipsychotic* or (neuroleptic adj3 (agent* or drug*)) or serotonin uptake inhibitor* or serotonin reuptake inhibitor* or ssri* or benzodiazepine* or (sedative adj3 (effect* or agent*)) or memantine or donepezil or rivastigmine or galantamine or souvenaid or risperidone or haloperidol or olanzapine or quetiapine or citalopram or dextromethorphan or carbamazepine or mirtazapine or sertraline or moclobemide or trazodone or melatonin or ramelteon or methylphenidate or exercis* or physical activit* or physical training or aerobic* or arobic* or kinesi?therap* or kinesi? therap* or (social adj3 (activit* or engag* or stimul*)) or psycholog* therap* or psychotherap* or ((behavio?r* or conditioning) adj3 therap*) or counsel?ing or ((psychosocial or psycho social) adj3 (support or interven* or care)) or ((alternative or compl?ment* or traditional) adj3 (medicine* or therap*)) or acupunct* or (herb* adj3 (tea* or remedy or remedies or medicine*)) or gingko or homeopath* or ((music or art or aroma or light or photo or pet or pets) adj3 therap*) or aromatherap* or phototherap* or massage* or (mind adj3 body) or (advance? adj3 (care or medical or healthcare) adj3 plan*) or (decision* adj3 (aid* or support)) or (communicati* adj3 skill* adj3 training) or (dementia care adj3 map*) or ((person* or patient*) adj3 cent* adj3 care) or "Resources for Enhancing Alzheimer’s Caregiver Health" or "Strategies for Relatives" or ((caregiver* or carer*) adj3 educat*) or telemedicine or tele medicine or (smart adj2 (phone* or device* or tablet*)) or smartphone* or cognitive aid* or reminder* or robot*).ti,ab. |
| 3 | Randomized Controlled Trials/ or (randomi#ed or placebo or randomly or trial or groups).ti,ab. |
| 4 | (Low Income Countries or Lower-Middle Income Countries or Least Developed Countries).sh. or (Africa* or Asia* or Caribbean* or West Indies or South America* or Latin America* or Central America* or Afghan* or Albania* or Algeria* or Angola* or Anguilla* or Antigua* or Barbuda* or Argentin* or Armenia* or Azerbaijan* or Azeri or Bangladesh* or Barbad* or Benin* or Byelarus or Byelorussian or Belarus* or Belorussia* or Belize* or Bhutan* or Bolivia* or Bosnia* or Herzegovin* or Hercegovin* or Botswana or Botsuana or Motswana or Batswana or Brasil* or Brazil* or Burkina Faso or Burkina Fasso or Burkina* or Burundi* or Urundi* or Cambodia* or Cameroon* or Cameron* or Cape Verd* or Cabo Verde or Central African Republic or Chad* or Tchad* or Chile* or China or Chinese or Colombia* or Columbia* or Comoros or Comoro Islands or Comores or Comoran or Mayotte or Congo* or Costa Rica* or Cote d'Ivoire or Ivory Coast or Ivorian* or Cook Islands or Cuba* or Croat* or Djibouti* or Dominica* or East Timor or East Timur or Timor Leste or Timorese or Ecuador* or Equador* or Egypt* or El Salvador or Salvadoran or Eritrea* or Ethiopia* or Fiji* or Gabon* or Gambia* or Gaza or Georgia Republic or Georgian or Abkhazia* or Abchasia* or South Ossetia* or Ghana* or Grenada or Grenadian or Guatemala* or Guinea* or Guinea Bissau or Guian* or Guyana or Haiti* or Hondura* or India or Indian or Indonesia* or Iran* or Iraq* or Jamaica* or Jordan* or Kazakhstan* or Kazakh or Kenya* or Kiribati or Korea* or Kosovo or Kosova* or Kyrgyzstan or Kirghizia or Kyrgyz or Kirghiz or Kirgizstan or Lao PDR or Laos or Laotian or Lebanon or Lebanese or Lesotho or Mosotho or Basotho or Liberia* or Libya* or Macedonia* or FYROM or Madagasca* or Malagasy or Malaysia* or Malaya* or Malay or Sabah or Sarawak or Malawi* or Maldives or Maldivan or Mali or Malian or Marshall Islands or Marshallese or Mauritania* or Mauriti* or Agalega Islands or Mexico or Mexican or Micronesia* or Middle East* or Moldova* or Moldovia* or Transnistria* or Mongolia* or Montenegr* or Montserrat* or Morocc* or Mozambique or Mozambican or Myanmar* or Myanma or Burma or Burmese or Namibia* or Nauru* or Niue or Nepal* or Nicaragua* or Niger or Nigerien or Nigeria* or Oman* or Pakistan* or Palau* or Palestine or Palestinian or Panama or Panamanian or Paraguay* or Papua New Guinea* or Peru or Peruvian or Philippines or Philipines or Phillipines or Phillippines or Filipino or Philipino or Philippino or Phillipino or Phillippino or Rwanda* or Ruanda* or Saint Helen* or St Helen* or Saint Kitts or St Kitts or Kittian or Nevis* or Saint Lucia* or St Lucia* or Saint Vincent or St Vincent or Vicentian or Grenadines or Samoa* or Sao Tome* or Senegal* or Serbia* or Seychell* or Sierra Leone* or Sri Lanka* or Ceylon or Solomon Island* or Somali* or South Africa* or Sudan or Sudanese or Surinam* or Swaziland or Swazi or Eswatini or Syria or Syrian or Tajikistan or Tadzhikistan or Tadjikistan or Tajik or Tadzhik or Tanzania* or Thailand or Thai or Togo or Togolese or Tonga* or Tunisia* or Tokelau or Trinidad* or Tobago* or Turkey or Turkish or Turks or Turkmenistan or Turkmen or Tuvalu* or Uganda* or Ukraine or Ukrainian or Uruguay* or Uzbekistan* or Uzbek or Vanuatu or Venezuela* or Vietnam* or Viet Nam or Wallis Futuna or West Bank or Yemen* or Zambia* or Zimbabwe*).hw,ti,ab,cp. or (((developing or less* developed or under developed or underdeveloped or middle income or low* income or underserved or under served or deprived or poor*) adj (countr* or nation? or population? or world)) or ((developing or less* developed or under developed or underdeveloped or middle income or low* income) adj (economy or economies)) or (low* adj (gdp or gnp or gross domestic or gross national)) or (low adj3 middle adj3 countr*) or lmic or lmics or third world or lami countr* or transitional countr*).ti,ab. |
| 5 | 1 and 2 and 3 and 4 |
| 6 | limit 5 to yr="2019 - 2020" |
| APA PsycInfo <1806 to September Week 1 2022> | |
| Condition | |
| 1 | exp Dementia/ or Huntington Disease/ or Cognitive Impairment/ or (dement* or amentia* or (major adj3 cognit* adj3 disorder*) or alzheimer* or alzeimer* or (cortical adj4 sclerosis) or ((encephalopath* or cogniti* or neurocogniti*) adj4 (aids or acquired immun?deficiency syndrome* or acquired immun? deficiency syndrome* or hiv or human immun?deficiency virus* or human immun? deficiency virus*)) or pick* disease* or (lobar adj3 atroph* adj3 brain) or Huntington* disease* or Huntington* chorea or (Lewy bod* adj3 disease*) or (cerebr* adj3 deteriorat*) or (cerebr* adj3 insufficien*) or ((frontotemporal or fronto temporal or corticobasal or cortico basal or frontal lobe*) adj4 (degenerati* or dysfunction*)) or ((cognit* or memory or cerebr*) adj3 (declin* or impair* or los* or deteriorat* or degenerat* or insufficen*)) or MCI or (mild adj2 cognit* impair*)).ti,ab. |
| 2 | exp Cognitive Therapy/ or exp Drug Therapy/ or exp Cholinesterase Inhibitors/ or exp Tranquilizing Drugs/ or exp Serotonin Reuptake Inhibitors/ or exp Benzodiazepines/ or exp Sedatives/ or exp Exercise/ or Movement Therapy/ or exp Psychotherapy/ or exp Counseling/ or Social Support/ or exp Alternative Medicine/ or Creative Arts Therapy/ or Mind Body Therapy/ or Phototherapy/ or Advance Directives/ or exp Case Management/ or Support Groups/ or exp Self-Help Techniques/ or exp Social Support/ or Computer Assisted Diagnosis/ or Computer Assisted Therapy/ or Telemedicine/ or exp Mobile Devices/ or (intervention* or therap* or treatment* or program* or manage* or prevent* or diagnos* or polic* or (cognit* adj3 (therap* or training or rehab*)) or cognit* psycho therap* or cognit* psychotherap* or drug* or medicine* or pharmacotherap* or pharmaco* therap* or cholinesterase agent* or cholinesterase inhibitor* or (tranquili* adj3 (agent* or drug*)) or antipsychotic* or (neuroleptic adj3 (agent* or drug*)) or serotonin uptake inhibitor* or serotonin reuptake inhibitor* or ssri* or benzodiazepine* or (sedative adj3 (effect* or agent*)) or memantine or donepezil or rivastigmine or galantamine or souvenaid or risperidone or haloperidol or olanzapine or quetiapine or citalopram or dextromethorphan or carbamazepine or mirtazapine or sertraline or moclobemide or trazodone or melatonin or ramelteon or methylphenidate or exercis* or physical activit* or physical training or aerobic* or arobic* or kinesi?therap* or kinesi? therap* or (social adj3 (activit* or engag* or stimul*)) or psycholog* therap* or psychotherap* or ((behavio?r* or conditioning) adj3 therap*) or counsel?ing or ((psychosocial or psycho social) adj3 (support or interven* or care)) or ((alternative or compl?ment* or traditional) adj3 (medicine* or therap*)) or acupunct* or (herb* adj3 (tea* or remedy or remedies or medicine*)) or gingko or homeopath* or ((music or art or aroma or light or photo or pet or pets) adj3 therap*) or aromatherap* or phototherap* or massage* or (mind adj3 body) or (advance? adj3 (care or medical or healthcare) adj3 plan*) or (decision* adj3 (aid* or support)) or (communicati* adj3 skill* adj3 training) or (dementia care adj3 map*) or ((person* or patient*) adj3 cent* adj3 care) or "Resources for Enhancing Alzheimer’s Caregiver Health" or "Strategies for Relatives" or ((caregiver* or carer*) adj3 educat*) or telemedicine or tele medicine or (smart adj2 (phone* or device* or tablet*)) or smartphone* or cognitive aid* or reminder* or robot*).ti,ab. |
| 3 | exp Clinical Trials/ or Treatment Effectiveness Evaluation/ or (randomi#ed or placebo or randomly or trial or groups).ti,ab. |
| 4 | Developing Countries.sh. or (Africa* or Asia* or Caribbean* or West Indies or South America* or Latin America* or Central America* or Afghan* or Albania* or Algeria* or Angola* or Anguilla* or Antigua* or Barbuda* or Argentin* or Armenia* or Azerbaijan* or Azeri or Bangladesh* or Barbad* or Benin* or Byelarus or Byelorussian or Belarus* or Belorussia* or Belize* or Bhutan* or Bolivia* or Bosnia* or Herzegovin* or Hercegovin* or Botswana or Botsuana or Motswana or Batswana or Brasil* or Brazil* or Burkina Faso or Burkina Fasso or Burkina* or Burundi* or Urundi* or Cambodia* or Cameroon* or Cameron* or Cape Verd* or Cabo Verde or Central African Republic or Chad* or Tchad* or Chile* or China or Chinese or Colombia* or Columbia* or Comoros or Comoro Islands or Comores or Comoran or Mayotte or Congo* or Costa Rica* or Cote d'Ivoire or Ivory Coast or Ivorian* or Cook Islands or Cuba* or Croat* or Djibouti* or Dominica* or East Timor or East Timur or Timor Leste or Timorese or Ecuador* or Equador* or Egypt* or El Salvador or Salvadoran or Eritrea* or Ethiopia* or Fiji* or Gabon* or Gambia* or Gaza or Georgia Republic or Georgian or Abkhazia* or Abchasia* or South Ossetia* or Ghana* or Grenada or Grenadian or Guatemala* or Guinea* or Guinea Bissau or Guian* or Guyana or Haiti* or Hondura* or India or Indian or Indonesia* or Iran* or Iraq* or Jamaica* or Jordan* or Kazakhstan* or Kazakh or Kenya* or Kiribati or Korea* or Kosovo or Kosova* or Kyrgyzstan or Kirghizia or Kyrgyz or Kirghiz or Kirgizstan or Lao PDR or Laos or Laotian or Lebanon or Lebanese or Lesotho or Mosotho or Basotho or Liberia* or Libya* or Macedonia* or FYROM or Madagasca* or Malagasy or Malaysia* or Malaya* or Malay or Sabah or Sarawak or Malawi* or Maldives or Maldivan or Mali or Malian or Marshall Islands or Marshallese or Mauritania* or Mauriti* or Agalega Islands or Mexico or Mexican or Micronesia* or Middle East* or Moldova* or Moldovia* or Transnistria* or Mongolia* or Montenegr* or Montserrat* or Morocc* or Mozambique or Mozambican or Myanmar* or Myanma or Burma or Burmese or Namibia* or Nauru* or Niue or Nepal* or Nicaragua* or Niger or Nigerien or Nigeria* or Oman* or Pakistan* or Palau* or Palestine or Palestinian or Panama or Panamanian or Paraguay* or Papua New Guinea* or Peru or Peruvian or Philippines or Philipines or Phillipines or Phillippines or Filipino or Philipino or Philippino or Phillipino or Phillippino or Rwanda* or Ruanda* or Saint Helen* or St Helen* or Saint Kitts or St Kitts or Kittian or Nevis* or Saint Lucia* or St Lucia* or Saint Vincent or St Vincent or Vicentian or Grenadines or Samoa* or Sao Tome* or Senegal* or Serbia* or Seychell* or Sierra Leone* or Sri Lanka* or Ceylon or Solomon Island* or Somali* or South Africa* or Sudan or Sudanese or Surinam* or Swaziland or Swazi or Eswatini or Syria or Syrian or Tajikistan or Tadzhikistan or Tadjikistan or Tajik or Tadzhik or Tanzania* or Thailand or Thai or Togo or Togolese or Tonga* or Tunisia* or Tokelau or Trinidad* or Tobago* or Turkey or Turkish or Turks or Turkmenistan or Turkmen or Tuvalu* or Uganda* or Ukraine or Ukrainian or Uruguay* or Uzbekistan* or Uzbek or Vanuatu or Venezuela* or Vietnam* or Viet Nam or Wallis Futuna or West Bank or Yemen* or Zambia* or Zimbabwe*).hw,ti,ab,lo. or (((developing or less* developed or under developed or underdeveloped or middle income or low* income or underserved or under served or deprived or poor*) adj (countr* or nation? or population? or world)) or ((developing or less* developed or under developed or underdeveloped or middle income or low* income) adj (economy or economies)) or (low* adj (gdp or gnp or gross domestic or gross national)) or (low adj3 middle adj3 countr*) or lmic or lmics or third world or lami countr* or transitional countr*).ti,ab. |
| 5 | 1 and 2 and 3 and 4 |
| 6 | (animal not human).po. |
| 7 | 5 not 6 |
| 8 | limit 7 to yr="2019 - 2020" |
| CINAHL EBSCO | |
| Condition | |
| S1 | (MH "Dementia+") or (MH "Huntington's Disease") or (MH "Cognition Disorders") or TI ( (dement* or amentia* or (major N2 cognit* N2 disorder*) or alzheimer* or alzeimer* or (cortical N3 sclerosis) or ((encephalopath* or cogniti* or neurocogniti*) N3 (aids or "acquired immun#deficiency syndrome*" or "acquired immun# deficiency syndrome*" or hiv or "human immun#deficiency virus*" or "human immun# deficiency virus*")) or "pick* disease*" or (lobar N2 atroph* N2 brain) or "Huntington* disease*" or "Huntington* chorea" or ("Lewy bod*" N2 disease*) or (cerebr* N2 deteriorat*) or (cerebr* N2 insufficien*) or ((frontotemporal or "fronto temporal" or corticobasal or "cortico basal" or "frontal lobe*") N3 (degenerati* or dysfunction*)) or ((cognit* or memory or cerebr*) N2 (declin* or impair* or los* or deteriorat* or degenerat* or insufficen*)) or MCI or (mild N1 "cognit* impair*")) ) OR AB ( (dement* or amentia* or (major N2 cognit* N2 disorder*) or alzheimer* or alzeimer* or (cortical N3 sclerosis) or ((encephalopath* or cogniti* or neurocogniti*) N3 (aids or "acquired immun#deficiency syndrome*" or "acquired immun# deficiency syndrome*" or hiv or "human immun#deficiency virus*" or "human immun# deficiency virus*")) or "pick* disease*" or (lobar N2 atroph* N2 brain) or "Huntington* disease*" or "Huntington* chorea" or ("Lewy bod*" N2 disease*) or (cerebr* N2 deteriorat*) or (cerebr* N2 insufficien*) or ((frontotemporal or "fronto temporal" or corticobasal or "cortico basal" or "frontal lobe*") N3 (degenerati* or dysfunction*)) or ((cognit* or memory or cerebr*) N2 (declin* or impair* or los* or deteriorat* or degenerat* or insufficen*)) or MCI or (mild N1 "cognit* impair*")) ) |
| S2 | (MH "Cognitive Therapy+") or (MH "Drug Therapy+") or (MH "Cholinesterase Inhibitors+") or (MH "Antipsychotic Agents+") or (MH "Serotonin Uptake Inhibitors+") or (MH "Antianxiety Agents, Benzodiazepine+") or (MH "Hypnotics and Sedatives+") or (MH "Exercise+") or (MH "Therapeutic Exercise+") or (MH "Psychotherapy+") or (MH "Counseling+") or (MH "Support, Psychosocial+") or (MH "Alternative Therapies+") or (MH "Advance Care Planning") or (MH "Advance Directives+") or (MH "Case Management") or (MH "Patient Care Plans+") or (MH "Caregivers/ED/PF") or (MH "Support Groups") or (MH "Diagnosis, Computer Assisted") or (MH "Telehealth+") or (MH "Computers, Hand-Held+") or TI ( (intervention* or therap* or treatment* or program* or manage* or prevent* or diagnos* or polic* or (cognit* N2 (therap* or training or rehab*)) or "cognit* psycho therap*" or "cognit* psychotherap*" or drug* or medicine* or pharmacotherap* or "pharmaco* therap*" or "cholinesterase agent*" or "cholinesterase inhibitor*" or (tranquili* N2 (agent* or drug*)) or antipsychotic* or (neuroleptic N2 (agent* or drug*)) or "serotonin uptake inhibitor*" or "serotonin reuptake inhibitor*" or ssri* or benzodiazepine* or (sedative N2 (effect* or agent*)) or memantine or donepezil or rivastigmine or galantamine or souvenaid or risperidone or haloperidol or olanzapine or quetiapine or citalopram or dextromethorphan or carbamazepine or mirtazapine or sertraline or moclobemide or trazodone or melatonin or ramelteon or methylphenidate or exercis* or "physical activit*" or "physical training" or aerobic* or arobic* or kinesi#therap* or "kinesi# therap*" or (social N2 (activit* or engag* or stimul*)) or "psycholog* therap*" or psychotherap* or ((behavio#r* or conditioning) N2 therap*) or counsel#ing or ((psychosocial or "psycho social") N2 (support or interven* or care)) or ((alternative or compl#ment* or traditional) N2 (medicine* or therap*)) or acupunct* or (herb* N2 (tea* or remedy or remedies or medicine*)) or gingko or homeopath* or ((music or art or aroma or light or photo or pet or pets) N2 therap*) or aromatherap* or phototherap* or massage* or (mind N2 body) or (advance# N2 (care or medical or healthcare) N2 plan*) or (decision* N2 (aid* or support)) or (communicati* N2 skill* N2 training) or ("dementia care" N2 map*) or ((person* or patient*) N2 cent* N2 care) or "Resources for Enhancing Alzheimer’s Caregiver Health" or "Strategies for Relatives" or ((caregiver* or carer*) N2 educat*) or telemedicine or "tele medicine" or (smart N2 (phone* or device* or tablet*)) or smartphone* or "cognitive aid*" or reminder* or robot*) ) OR AB ( (intervention* or therap* or treatment* or program* or manage* or prevent* or diagnos* or polic* or (cognit* N2 (therap* or training or rehab*)) or "cognit* psycho therap*" or "cognit* psychotherap*" or drug* or medicine* or pharmacotherap* or "pharmaco* therap*" or "cholinesterase agent*" or "cholinesterase inhibitor*" or (tranquili* N2 (agent* or drug*)) or antipsychotic* or (neuroleptic N2 (agent* or drug*)) or "serotonin uptake inhibitor*" or "serotonin reuptake inhibitor*" or ssri* or benzodiazepine* or (sedative N2 (effect* or agent*)) or memantine or donepezil or rivastigmine or galantamine or souvenaid or risperidone or haloperidol or olanzapine or quetiapine or citalopram or dextromethorphan or carbamazepine or mirtazapine or sertraline or moclobemide or trazodone or melatonin or ramelteon or methylphenidate or exercis* or "physical activit*" or "physical training" or aerobic* or arobic* or kinesi#therap* or "kinesi# therap*" or (social N2 (activit* or engag* or stimul*)) or "psycholog* therap*" or psychotherap* or ((behavio#r* or conditioning) N2 therap*) or counsel#ing or ((psychosocial or "psycho social") N2 (support or interven* or care)) or ((alternative or compl#ment* or traditional) N2 (medicine* or therap*)) or acupunct* or (herb* N2 (tea* or remedy or remedies or medicine*)) or gingko or homeopath* or ((music or art or aroma or light or photo or pet or pets) N2 therap*) or aromatherap* or phototherap* or massage* or (mind N2 body) or (advance# N2 (care or medical or healthcare) N2 plan*) or (decision* N2 (aid* or support)) or (communicati* N2 skill* N2 training) or ("dementia care" N2 map*) or ((person* or patient*) N2 cent* N2 care) or "Resources for Enhancing Alzheimer’s Caregiver Health" or "Strategies for Relatives" or ((caregiver* or carer*) N2 educat*) or telemedicine or "tele medicine" or (smart N2 (phone* or device* or tablet*)) or smartphone* or "cognitive aid*" or reminder* or robot*) ) |
| S3 | (MH "Randomized Controlled Trials") or (MH "Double-Blind Studies") or (MH "Single-Blind Studies") or (MH "Random Assignment") or (MH "Pretest-Posttest Design+") or (MH "Cluster Sample+") or TI ( randomised or randomized ) or AB random* or TI trial or (MH "Sample Size" and AB (assigned OR allocated OR control)) or (MH "Placebos") or (MH "Crossover Design") or (MH "Comparative Studies") or PT "randomized controlled trial" or AB ((control W5 group) or (cluster W3 RCT)) |
| S4 | (MH "Developing Countries") |
| S5 | TI ( Africa* or Asia* or Caribbean* or "West Indies" or "South America*" or "Latin America*" or "Central America*" or Afghan* or Albania* or Algeria* or Angola* or Anguilla* or Antigua* or Barbuda* or Argentin* or Armenia* or Azerbaijan* or Azeri or Bangladesh* or Barbad* or Benin* or Byelarus or Byelorussian or Belarus* or Belorussia* or Belize* or Bhutan* or Bolivia* or Bosnia* or Herzegovin* or Hercegovin* or Botswana or Botsuana or Motswana or Batswana or Brasil* or Brazil* or "Burkina Faso" or "Burkina Fasso" or Burkina* or Burundi* or Urundi* or Cambodia* or Cameroon* or Cameron* or "Cape Verd*" or "Cabo Verde" or "Central African Republic" or Chad* or Tchad* or Chile* or China or Chinese or Colombia* or Columbia* or Comoros or "Comoro Islands" or Comores or Comoran or Mayotte or Congo* or "Costa Rica*" or "Cote d'Ivoire" or "Ivory Coast" or Ivorian* or "Cook Islands" or Cuba* or Croat* or Djibouti* or Dominica* or "East Timor" or "East Timur" or "Timor Leste" or Timorese or Ecuador* or Equador* or Egypt* or "El Salvador" or Salvadoran or Eritrea* or Ethiopia* or Fiji* or Gabon* or Gambia* or Gaza or "Georgia Republic" or Georgian or Abkhazia* or Abchasia* or "South Ossetia*" or Ghana* or Grenada or Grenadian or Guatemala* or Guinea* or "Guinea Bissau" or Guian* or Guyana or Haiti* or Hondura* or India or Indian or Indonesia* or Iran* or Iraq* or Jamaica* or Jordan* or Kazakhstan* or Kazakh or Kenya* or Kiribati or Korea* or Kosovo or Kosova* or Kyrgyzstan or Kirghizia or Kyrgyz or Kirghiz or Kirgizstan or "Lao PDR" or Laos or Laotian or Lebanon or Lebanese or Lesotho or Mosotho or Basotho or Liberia* or Libya* or Macedonia* or FYROM or Madagasca* or Malagasy or Malaysia* or Malaya* or Malay or Sabah or Sarawak or Malawi* or Maldives or Maldivan or Mali or Malian or "Marshall Islands" or Marshallese or Mauritania* or Mauriti* or "Agalega Islands" or Mexico or Mexican or Micronesia* or "Middle East*" or Moldova* or Moldovia* or Transnistria* or Mongolia* or Montenegr* or Montserrat* or Morocc* or Mozambique or Mozambican or Myanmar* or Myanma or Burma or Burmese or Namibia* or Nauru* or Niue or Nepal* or Nicaragua* or Niger or Nigerien or Nigeria* or Oman* or Pakistan* or Palau* or Palestine or Palestinian or Panama or Panamanian or Paraguay* or "Papua New Guinea*" or Peru or Peruvian or Philippines or Philipines or Phillipines or Phillippines or Filipino or Philipino or Philippino or Phillipino or Phillippino or Rwanda* or Ruanda* or "Saint Helen*" or "St Helen*" or "Saint Kitts" or "St Kitts" or Kittian or Nevis* or "Saint Lucia*" or "St Lucia*" or "Saint Vincent" or "St Vincent" or Vicentian or Grenadines or Samoa* or "Sao Tome*" or Senegal* or Serbia* or Seychell* or "Sierra Leone*" or "Sri Lanka*" or Ceylon or "Solomon Island*" or Somali* or "South Africa*" or Sudan or Sudanese or Surinam* or Swaziland or Swazi or Eswatini or Syria or Syrian or Tajikistan or Tadzhikistan or Tadjikistan or Tajik or Tadzhik or Tanzania* or Thailand or Thai or Togo or Togolese or Tonga* or Tunisia* or Tokelau or Trinidad* or Tobago* or Turkey or Turkish or Turks or Turkmenistan or Turkmen or Tuvalu* or Uganda* or Ukraine or Ukrainian or Uruguay* or Uzbekistan* or Uzbek or Vanuatu or Venezuela* or Vietnam* or "Viet Nam" or "Wallis Futuna" or "West Bank" or Yemen* or Zambia* or Zimbabwe* ) OR AB ( Africa* or Asia* or Caribbean* or "West Indies" or "South America*" or "Latin America*" or "Central America*" or Afghan* or Albania* or Algeria* or Angola* or Anguilla* or Antigua* or Barbuda* or Argentin* or Armenia* or Azerbaijan* or Azeri or Bangladesh* or Barbad* or Benin* or Byelarus or Byelorussian or Belarus* or Belorussia* or Belize* or Bhutan* or Bolivia* or Bosnia* or Herzegovin* or Hercegovin* or Botswana or Botsuana or Motswana or Batswana or Brasil* or Brazil* or "Burkina Faso" or "Burkina Fasso" or Burkina* or Burundi* or Urundi* or Cambodia* or Cameroon* or Cameron* or "Cape Verd*" or "Cabo Verde" or "Central African Republic" or Chad* or Tchad* or Chile* or China or Chinese or Colombia* or Columbia* or Comoros or "Comoro Islands" or Comores or Comoran or Mayotte or Congo* or "Costa Rica*" or "Cote d'Ivoire" or "Ivory Coast" or Ivorian* or "Cook Islands" or Cuba* or Croat* or Djibouti* or Dominica* or "East Timor" or "East Timur" or "Timor Leste" or Timorese or Ecuador* or Equador* or Egypt* or "El Salvador" or Salvadoran or Eritrea* or Ethiopia* or Fiji* or Gabon* or Gambia* or Gaza or "Georgia Republic" or Georgian or Abkhazia* or Abchasia* or "South Ossetia*" or Ghana* or Grenada or Grenadian or Guatemala* or Guinea* or "Guinea Bissau" or Guian* or Guyana or Haiti* or Hondura* or India or Indian or Indonesia* or Iran* or Iraq* or Jamaica* or Jordan* or Kazakhstan* or Kazakh or Kenya* or Kiribati or Korea* or Kosovo or Kosova* or Kyrgyzstan or Kirghizia or Kyrgyz or Kirghiz or Kirgizstan or "Lao PDR" or Laos or Laotian or Lebanon or Lebanese or Lesotho or Mosotho or Basotho or Liberia* or Libya* or Macedonia* or FYROM or Madagasca* or Malagasy or Malaysia* or Malaya* or Malay or Sabah or Sarawak or Malawi* or Maldives or Maldivan or Mali or Malian or "Marshall Islands" or Marshallese or Mauritania* or Mauriti* or "Agalega Islands" or Mexico or Mexican or Micronesia* or "Middle East*" or Moldova* or Moldovia* or Transnistria* or Mongolia* or Montenegr* or Montserrat* or Morocc* or Mozambique or Mozambican or Myanmar* or Myanma or Burma or Burmese or Namibia* or Nauru* or Niue or Nepal* or Nicaragua* or Niger or Nigerien or Nigeria* or Oman* or Pakistan* or Palau* or Palestine or Palestinian or Panama or Panamanian or Paraguay* or "Papua New Guinea*" or Peru or Peruvian or Philippines or Philipines or Phillipines or Phillippines or Filipino or Philipino or Philippino or Phillipino or Phillippino or Rwanda* or Ruanda* or "Saint Helen*" or "St Helen*" or "Saint Kitts" or "St Kitts" or Kittian or Nevis* or "Saint Lucia*" or "St Lucia*" or "Saint Vincent" or "St Vincent" or Vicentian or Grenadines or Samoa* or "Sao Tome*" or Senegal* or Serbia* or Seychell* or "Sierra Leone*" or "Sri Lanka*" or Ceylon or "Solomon Island*" or Somali* or "South Africa*" or Sudan or Sudanese or Surinam* or Swaziland or Swazi or Eswatini or Syria or Syrian or Tajikistan or Tadzhikistan or Tadjikistan or Tajik or Tadzhik or Tanzania* or Thailand or Thai or Togo or Togolese or Tonga* or Tunisia* or Tokelau or Trinidad* or Tobago* or Turkey or Turkish or Turks or Turkmenistan or Turkmen or Tuvalu* or Uganda* or Ukraine or Ukrainian or Uruguay* or Uzbekistan* or Uzbek or Vanuatu or Venezuela* or Vietnam* or "Viet Nam" or "Wallis Futuna" or "West Bank" or Yemen* or Zambia* or Zimbabwe* ) OR MH ( Africa* or Asia* or Caribbean* or "West Indies" or "South America*" or "Latin America*" or "Central America*" or Afghan* or Albania* or Algeria* or Angola* or Anguilla* or Antigua* or Barbuda* or Argentin* or Armenia* or Azerbaijan* or Azeri or Bangladesh* or Barbad* or Benin* or Byelarus or Byelorussian or Belarus* or Belorussia* or Belize* or Bhutan* or Bolivia* or Bosnia* or Herzegovin* or Hercegovin* or Botswana or Botsuana or Motswana or Batswana or Brasil* or Brazil* or "Burkina Faso" or "Burkina Fasso" or Burkina* or Burundi* or Urundi* or Cambodia* or Cameroon* or Cameron* or "Cape Verd*" or "Cabo Verde" or "Central African Republic" or Chad* or Tchad* or Chile* or China or Chinese or Colombia* or Columbia* or Comoros or "Comoro Islands" or Comores or Comoran or Mayotte or Congo* or "Costa Rica*" or "Cote d'Ivoire" or "Ivory Coast" or Ivorian* or "Cook Islands" or Cuba* or Croat* or Djibouti* or Dominica* or "East Timor" or "East Timur" or "Timor Leste" or Timorese or Ecuador* or Equador* or Egypt* or "El Salvador" or Salvadoran or Eritrea* or Ethiopia* or Fiji* or Gabon* or Gambia* or Gaza or "Georgia Republic" or Georgian or Abkhazia* or Abchasia* or "South Ossetia*" or Ghana* or Grenada or Grenadian or Guatemala* or Guinea* or "Guinea Bissau" or Guian* or Guyana or Haiti* or Hondura* or India or Indian or Indonesia* or Iran* or Iraq* or Jamaica* or Jordan* or Kazakhstan* or Kazakh or Kenya* or Kiribati or Korea* or Kosovo or Kosova* or Kyrgyzstan or Kirghizia or Kyrgyz or Kirghiz or Kirgizstan or "Lao PDR" or Laos or Laotian or Lebanon or Lebanese or Lesotho or Mosotho or Basotho or Liberia* or Libya* or Macedonia* or FYROM or Madagasca* or Malagasy or Malaysia* or Malaya* or Malay or Sabah or Sarawak or Malawi* or Maldives or Maldivan or Mali or Malian or "Marshall Islands" or Marshallese or Mauritania* or Mauriti* or "Agalega Islands" or Mexico or Mexican or Micronesia* or "Middle East*" or Moldova* or Moldovia* or Transnistria* or Mongolia* or Montenegr* or Montserrat* or Morocc* or Mozambique or Mozambican or Myanmar* or Myanma or Burma or Burmese or Namibia* or Nauru* or Niue or Nepal* or Nicaragua* or Niger or Nigerien or Nigeria* or Oman* or Pakistan* or Palau* or Palestine or Palestinian or Panama or Panamanian or Paraguay* or "Papua New Guinea*" or Peru or Peruvian or Philippines or Philipines or Phillipines or Phillippines or Filipino or Philipino or Philippino or Phillipino or Phillippino or Rwanda* or Ruanda* or "Saint Helen*" or "St Helen*" or "Saint Kitts" or "St Kitts" or Kittian or Nevis* or "Saint Lucia*" or "St Lucia*" or "Saint Vincent" or "St Vincent" or Vicentian or Grenadines or Samoa* or "Sao Tome*" or Senegal* or Serbia* or Seychell* or "Sierra Leone*" or "Sri Lanka*" or Ceylon or "Solomon Island*" or Somali* or "South Africa*" or Sudan or Sudanese or Surinam* or Swaziland or Swazi or Eswatini or Syria or Syrian or Tajikistan or Tadzhikistan or Tadjikistan or Tajik or Tadzhik or Tanzania* or Thailand or Thai or Togo or Togolese or Tonga* or Tunisia* or Tokelau or Trinidad* or Tobago* or Turkey or Turkish or Turks or Turkmenistan or Turkmen or Tuvalu* or Uganda* or Ukraine or Ukrainian or Uruguay* or Uzbekistan* or Uzbek or Vanuatu or Venezuela* or Vietnam* or "Viet Nam" or "Wallis Futuna" or "West Bank" or Yemen* or Zambia* or Zimbabwe* ) |
| S6 | TI ( (developing or "less* developed" or "under developed" or underdeveloped or "middle income" or "low* income" or underserved or "under served" or deprived or poor*) N1 (countr* or nation# or population# or world)) or ((developing or "less* developed" or "under developed" or underdeveloped or "middle income" or "low* income") N1 (economy or economies)) or (low* N1 (gdp or gnp or "gross domestic" or "gross national")) or (low N2 middle N2 countr*) or lmic or lmics or "third world" or "lami countr*" or "transitional countr*" ) OR AB ( (developing or "less* developed" or "under developed" or underdeveloped or "middle income" or "low* income" or underserved or "under served" or deprived or poor*) N1 (countr* or nation# or population# or world)) or ((developing or "less* developed" or "under developed" or underdeveloped or "middle income" or "low* income") N1 (economy or economies)) or (low* N1 (gdp or gnp or "gross domestic" or "gross national")) or (low N2 middle N2 countr*) or lmic or lmics or "third world" or "lami countr*" or "transitional countr*" ) |
| S7 | S4 or S5 or S6 |
| S8 | S1 AND S2 AND S3 AND S7 |
| S9 | (MH "Animals+" OR MH "Animal Studies" OR TI "animal model*") NOT MH "Human" |
| S10 | S8 NOT S9 |
| S11 | Limiters - Published Date: 20190101-20211231 |

1. Characteristics of included trials
   1. Trends of dementia caregiver intervention trials in LMICs

- 1. Geographic distribution of included trials

- 1. Human development index (HDI) of trial locations in comparison with national HDI

- 1. Mean ages and female percentages of study cohorts

1. List of most measured outcomes and measurement instruments

| Effect measure | # of trials | Instrument (# of trials) |
| --- | --- | --- |
| Caregiver outcomes |  |  |
| perceived burden | 24 | ZBI (19), CBI (2), CBDPCS (1), CSI (1), Unclear (1) |
| depression | 10 | BDI (5), CESD-20 (2), CESD-10 (1), GHQ-28 (1), Ham-D (1) |
| quality of life | 9 | WHO-QoL-Bref (5), EuroQol-VAS (1), GQOLI-74 (1), LSS (1), QoL-ADc (1) |
| care-related distress | 8 | NPI-Q distress (8) |
| anxiety | 5 | BAI (2), GHQ-28 (1), Spielberger's (1), TMAS (1) |
| stress and distress | 5 | GHQ-12 (1), GHQ-28 (1), PHQ-4 (1), PHQ-9 (1), PSS (1), SRQ-20 (1) |
| physical quality of life | 4 | WHO-QoL-Bref (3), SF36 (1) |
| psychological quality of life | 4 | WHO-QoL-Bref (3), SF36 (1) |
| environmental quality of life | 3 | WHO-QoL-Bref (3) |
| knowledge of dementia | 3 | AD Knowledge (3) |
| social quality of life | 3 | WHO-QoL-Bref (3) |
| Care recipient outcomes |  |  |
| quality of life | 13 | QoL-AD (7), SF36 (3), DEMQOL (1), Kane’s (1), Unclear (1) |
| neuropsychiatric symptoms | 11 | NPI-Q severity (9), BEHAVE-AD (2) |
| cognitive function | 10 | MMSE (9), ADAS-Cog (1), MoCA (1) |
| composite activities of daily life^[[1]](#footnote-1)^ | 6 | Chinese ADL (5), Finch's (1) |
| agitation | 4 | CMAI (2), NPI-Q severity (2) |
| basic activities of daily life | 4 | Barthel Index (2), ADLQ (1), PSMS (1) |
| depression | 3 | NPI-Q severity (2), Cornell (1) |

**Abbreviations:** AD Knowledge, Alzheimer's Disease Knowledge Scale; ADAS-cog, Alzheimer's Disease Assessment Scale-Cognitive Subscale; ADL, Activities of daily living; ADLQ, Activities of Daily Living Questionnaire; BAI, Beck Anxiety Inventory; Barthel Index, Barthel Index of Activities of Daily Living; BDI, Beck Depression Inventory; BEHAVE-AD, Behaviour Pathology in Alzheimer's Disease Rating Scale; CBDPCS, Caregiver Burden of Dementia Patient Caregiver Scale; CBI, Caregiver burden Inventory; CESD-10, 10-item Center for Epidemiologic Studies Depression Scale; CESD-20, 20-item Center for Epidemiologic Studies Depression Scale; Chinese ADL, Activity of Daily Living Scale (Chinese version); CMAI, Cohen-Mansfield Agitation Inventory; Cornell, Cornell Scale for Depression in Dementia; CSI, Caregiver Strain Index; DEMQOL, Dementia Quality of Life measure; EuroQol-VAS, EuroQol visual analogue scale; Finch's, Finch's ADL scale; MMSE, Mini-Mental State Examination; GHQ-12, 12-Item General Health Questionnaire; GHQ-28, 28-item General Health Questionnaire; GQOLI-74, 74-item Generic quality of life inventory; Ham-D, Hamilton Depression Rating Scale; Kane's, Kane's Quality of Life Scale; LSS, Life Situation Survey; MoCA, Montreal Cognitive Assessment; NPI-D, Neuropsychiatric Symptoms Inventory (distress subscale); NPI-S, Neuropsychiatric Symptoms Inventory (severity subscale); PHQ-4, 4-item Patient Health Questionnaire; PHQ-9, 9-item Patient Health Questionnaire; PSMS, Physical Self-maintenance Scale; PSS, Perceived Stress Scale; QoL-AD, Quality of Life in Alzheimer’s disease; QoL-ADc, Quality of Life in Alzheimer's Disease scale (Carer Version); SF36, 36-Item Short Form Survey; Spielberger's, Spielberger’s questionnaires; SRQ-20, 20-item self-report screening tool; TMAS, Taylor Manifest Anxiety Scale; WHO-Bref-QoL, World Health Organization Quality-of-Life Scale; ZBI, Zarit Burden Interview.

1. Selected characteristics of participants in included trials

| Study ID | Group | Source | Inclusion criteria | | Dementia patient | | Carers | |  | Total sample | |
| --- | --- | --- | --- | --- | --- | --- | --- | --- | --- | --- | --- |
|  |  |  | **Type of disease^[[2]](#footnote-2)^** | **Severity** | **Female %** | **Age** | **Female %** | **Age** |  | **N** | **Attrition %** |
| Arango-Lasprilla 2014 | Control | Patient registration | Dementia | Not specified |  |  | 73.30 | 55.10 |  |  |  |
| Arango-Lasprilla 2014 | Intervention | Patient registration | Dementia | Not specified |  |  | 87.20 | 59.40 |  |  |  |
| Aslan 2022 | Intervention | Hospital admission | Dementia | Any severity stage | 50.00 | 77.44 | 68.60 | 48.89 |  |  |  |
| Aslan 2022 | Control | Hospital admission | Dementia | Any severity stage | 42.90 | 77.61 | 80.00 | 52.17 |  |  |  |
| Baruah 2021 | Intervention | Community | Alzheimer’s Disease | Not specified | 76.40 | 74.62 | 74.50 | 53.03 |  | 74 | 60.81 |
| Baruah 2021 | Control | Community | Alzheimer’s Disease | Not specified | 74.50 | 70.35 | 74.50 | 45.88 |  | 75 | 68.00 |
| Chen 2020 | Control | Hospital admission | Alzheimer’s Disease | Moderate to Severe | 52.90 | 71.43 |  |  |  |  |  |
| Chen 2020 | Intervention | Hospital admission | Alzheimer’s Disease | Moderate to Severe | 52.80 | 72.38 |  |  |  |  |  |
| Danucalov 2013 | Intervention | Community | Alzheimer's Disease | Not specified |  |  | 88.00 | 55.50 |  |  |  |
| Danucalov 2013 | Control | Community | Alzheimer's Disease | Not specified |  |  | 90.00 | 53.40 |  |  |  |
| Dias 2008 | Intervention | Community | Dementia | Mild to moderate | 36.60 | 79.40 | 90.20 | 53.20 |  | 41 | 19.51 |
| Dias 2008 | Control | Community | Dementia | Mild to moderate | 32.50 | 77.30 | 85.00 | 53.80 |  | 40 | 35.00 |
| Duru Asiret 2021 | Intervention | Hospital admission | Dementia | Moderate to Severe | 73.30 | 81.73 | 93.30 | 48.13 |  | 18 | 16.67 |
| Duru Asiret 2021 | Control | Hospital admission | Dementia | Moderate to Severe | 78.60 | 82.42 | 100.00 | 45.42 |  | 18 | 22.22 |
| Ghaffari 2019 | Control | Hospital admission | Mild cognitive impairment | Mild |  |  | 72.00 | 43.40 |  | 27 | 7.41 |
| Ghaffari 2019 | Intervention | Hospital admission | Mild cognitive impairment | Mild |  |  | 88.00 | 42.60 |  | 27 | 7.41 |
| Gok Ugur 2018 | Control | Hospital admission | Dementia | Not specified | 80.00 | 83.40 | 86.70 | 54.60 |  |  |  |
| Gok Ugur 2018 | Intervention | Hospital admission | Dementia | Not specified | 76.70 | 82.43 | 93.30 | 50.43 |  |  |  |
| Govindakumari 2020 | Intervention | Community | Mild dementia | Mild |  |  |  |  |  |  |  |
| Govindakumari 2020 | Control | Community | Mild dementia | Mild |  |  |  |  |  |  |  |
| Guerra 2011 | Intervention | Community | Dementia | Any severity stage | 79.30 | 81.70 | 85.70 | 53.30 |  | 29 | 6.90 |
| Guerra 2011 | Control | Community | Dementia | Any severity stage | 69.00 | 82.00 | 89.70 | 47.60 |  | 29 | 0.00 |
| He 2012 | Control | Hospital admission | Dementia | Mild to moderate | 47.10 | 75.90 |  |  |  |  |  |
| He 2012 | Intervention | Hospital admission | Dementia | Mild to moderate | 41.20 | 76.80 |  |  |  |  |  |
| Heydari 2017 | Control | Hospital admission | Alzheimer's Disease | Not specified |  |  | 75.00 | 47.13 |  |  |  |
| Heydari 2017 | Intervention | Hospital admission | Alzheimer's Disease | Not specified |  |  | 55.56 | 52.44 |  |  |  |
| Hinton 2020 | Control | Community | Alzheimer’s Disease | Mild to moderate | 76.90 | 82.80 | 80.80 | 58.70 |  | 30 | 13.33 |
| Hinton 2020 | Intervention | Community | Alzheimer’s Disease | Mild to moderate | 76.00 | 83.70 | 92.00 | 59.00 |  | 30 | 16.67 |
| Jahani 2022 | Control | Hospital admission | Dementia | Not specified |  |  | 88.57 | 43.35 |  |  |  |
| Jahani 2022 | Intervention | Hospital admission | Dementia | Not specified |  |  | 77.14 | 45.48 |  |  |  |
| JIANG 2012 | Control | Hospital admission | Dementia | Mild to moderate | 54.17 | 71.33 |  |  |  | 48 | 0.00 |
| JIANG 2012 | Intervention | Hospital admission | Dementia | Mild to moderate | 58.33 | 71.94 |  |  |  | 48 | 4.17 |
| Kamkhagi 2015 | BAT group | Hospital admission | Dementia | Mild to moderate |  |  | 70.00 | 55.70 |  |  |  |
| Kamkhagi 2015 | PGT group | Hospital admission | Dementia | Mild to moderate |  |  | 75.00 | 62.10 |  |  |  |
| Liu 2017 | Control | Community | Dementia | Any severity stage | 64.10 |  | 56.41 | 62.79 |  | 39 | 0.00 |
| Liu 2017 | Intervention | Community | Dementia | Any severity stage | 64.10 |  | 66.67 | 66.54 |  | 39 | 0.00 |
| Lök 2017 | Control | Patient registration | Dementia | Not specified | 35.00 | 74.16 | 65.00 | 51.76 |  |  |  |
| Lök 2017 | Intervention | Patient registration | Dementia | Not specified | 45.00 | 70.65 | 70.00 | 52.97 |  |  |  |
| Lök 2019 | Intervention | Nursing home | Alzheimer’s Disease | Mild to moderate | 60.00 |  |  |  |  | 30 | 0.00 |
| Lök 2019 | Control | Nursing home | Alzheimer’s Disease | Mild to moderate | 53.40 |  |  |  |  | 30 | 0.00 |
| Mahdavi 2017 | Control 2 (No intervention) | Patient registration | Dementia | Not specified |  | 75.44 |  | 52.96 |  | 35 | 11.43 |
| Mahdavi 2017 | Control 1 (group sessions) | Patient registration | Dementia | Not specified |  | 75.44 |  | 52.96 |  | 32 | 12.50 |
| Mahdavi 2017 | Intervention | Patient registration | Dementia | Not specified |  | 75.44 |  | 52.96 |  | 33 | 9.09 |
| Novelli 2018 | Intervention | Community | Dementia | Not specified | 46.66 | 79.40 | 93.33 | 64.33 |  | 17 | 11.76 |
| Novelli 2018 | Control | Community | Dementia | Not specified | 53.33 | 83.49 | 73.33 | 68.16 |  | 15 | 0.00 |
| Oliveira 2018 | Control | Hospital admission | Dementia | Moderate | 50.00 | 78.40 | 90.00 | 60.70 |  |  |  |
| Oliveira 2018 | Intervention | Hospital admission | Dementia | Moderate | 90.00 | 79.00 | 63.60 | 56.80 |  |  |  |
| Oliveira 2021 | Control | Hospital admission | Dementia | Not specified | 61.50 | 76.30 | 76.90 | 60.00 |  |  |  |
| Oliveira 2021 | Intervention | Hospital admission | Dementia | Not specified | 71.40 | 78.40 | 78.60 | 54.00 |  |  |  |
| Pahlavanzadeh 2010 | Intervention | Hospital admission | Dementia | Not specified | 44.00 | 72.00 | 84.00 | 42.88 |  | 30 | 16.67 |
| Pahlavanzadeh 2010 | Control | Hospital admission | Dementia | Not specified | 56.00 | 67.72 | 68.00 | 46.56 |  | 30 | 16.67 |
| Pan 2019 | Intervention | Hospital admission | Dementia | Moderate to Severe | 71.43 | 79.00 | 55.36 | 63.27 |  | 56 | 16.07 |
| Pan 2019 | Control | Hospital admission | Dementia | Moderate to Severe | 63.39 | 79.96 | 69.64 | 62.09 |  | 56 | 37.50 |
| Pankong 2018 | Control | Hospital admission | Dementia | Mild to moderate |  |  | 72.20 | 55.66 |  | 40 | 10.00 |
| Pankong 2018 | Intervention | Hospital admission | Dementia | Mild to moderate |  |  | 83.30 | 55.11 |  | 40 | 10.00 |
| Serrani Azcurra 2012 | Intervention | Nursing home | Dementia | Mild to moderate | 66.50 | 85.30 |  |  |  |  |  |
| Serrani Azcurra 2012 | Passive control | Nursing home | Dementia | Mild to moderate | 65.50 | 85.80 |  |  |  |  |  |
| Serrani Azcurra 2012 | Active control | Nursing home | Dementia | Mild to moderate | 60.00 | 86.40 |  |  |  |  |  |
| Shata 2017 | Control | Hospital admission | Dementia | Not specified | 67.50 | 69.29 | 69.50 | 47.97 |  | 60 | 1.67 |
| Shata 2017 | Intervention | Hospital admission | Dementia | Not specified | 67.50 | 69.29 | 61.80 | 49.35 |  | 60 | 8.33 |
| Söylemez 2016 | Intervention | Hospital admission | Dementia | Mild | 63.00 | 78.60 | 74.00 | 61.20 |  | 35 | 5.71 |
| Söylemez 2016 | Control | Hospital admission | Dementia | Mild | 54.00 | 76.29 | 71.00 | 62.28 |  | 35 | 8.57 |
| SU 2012 | Control | Hospital admission | Dementia | Not specified | 55.00 | 68.00 |  |  |  |  |  |
| SU 2012 | Intervention | Hospital admission | Dementia | Not specified | 50.00 | 68.00 |  |  |  |  |  |
| SUN 2010 | Control | Hospital admission | Dementia | Mild to moderate | 52.63 | 73.20 |  |  |  |  |  |
| SUN 2010 | Intervention | Hospital admission | Dementia | Mild to moderate | 47.37 | 74.10 |  |  |  |  |  |
| TAN 2010 | Control | Hospital admission | Dementia | Mild to moderate | 61.53 | 68.43 |  |  |  |  |  |
| TAN 2010 | Intervention | Hospital admission | Dementia | Mild to moderate | 61.53 | 68.43 |  |  |  |  |  |
| Tawfik 2021 | Intervention | Hospital admission | Dementia | Mild to moderate | 33.30 | 69.90 | 86.70 | 37.40 |  | 34 | 11.76 |
| Tawfik 2021 | Control | Hospital admission | Dementia | Mild to moderate | 30.00 | 67.80 | 73.30 | 38.30 |  | 34 | 11.76 |
| Turten Kaymaz 2017 | Intervention | Hospital admission | Dementia | Not specified | 71.40 | 82.43 | 92.90 |  |  |  |  |
| Turten Kaymaz 2017 | Control | Hospital admission | Dementia | Not specified | 57.10 | 73.79 | 85.70 |  |  |  |  |
| Uyar 2019 | Control | Hospital admission | Alzheimer’s Disease | Any severity stage | 67.74 | 76.70 | 74.23 | 53.60 |  | 31 | 9.68 |
| Uyar 2019 | Intervention | Hospital admission | Alzheimer’s Disease | Any severity stage | 76.67 | 76.70 | 83.33 | 53.60 |  | 30 | 13.33 |
| Wang 2010 | Intervention | Community | Dementia | Mild to moderate |  |  |  |  |  |  |  |
| Wang 2010 | Control | Community | Dementia | Mild to moderate |  |  |  |  |  |  |  |
| Wang 2012 | Control | Hospital admission | Dementia | Any severity stage | 53.80 |  | 64.10 |  |  |  |  |
| Wang 2012 | Intervention | Hospital admission | Dementia | Any severity stage | 51.30 |  | 58.97 |  |  |  |  |
| Wang 2014 | Intervention | Community | Dementia | Not specified |  |  |  |  |  | 64 | 3.13 |
| Wang 2014 | Control | Community | Dementia | Not specified |  |  |  |  |  | 64 | 4.69 |
| Wang 2017c | Control | N/A | Dementia | Not specified |  |  | 93.44 | 31.9 |  | 54 | 3.70 |
| Wang 2017c | Intervention | N/A | Dementia | Not specified |  |  | 94.44 | 28.8 |  | 61 | 19.67 |
| Wang 2017e | Control | N/A | Dementia | Not specified |  |  | 83.50 | 31.30 |  | 92 | 7.61 |
| Wang 2017e | Intervention | N/A | Dementia | Not specified |  |  | 82.40 | 30.55 |  | 90 | 5.56 |
| Wang 2021 | Control | Hospital admission | Dementia | Moderate to Severe |  |  | 63.30 | 60.14 |  |  |  |
| Wang 2021 | Intervention | Hospital admission | Dementia | Moderate to Severe |  |  | 60.00 | 63.20 |  |  |  |
| Xu 2022 | Intervention | Hospital admission | Dementia | Moderate to Severe |  |  | 77.14 | 54.71 |  | 36 | 22.22 |
| Xu 2022 | Control | Hospital admission | Dementia | Moderate to Severe |  |  | 72.22 | 53.44 |  | 36 | 13.89 |
| Yang 2017 | Intervention | Community | Dementia | Moderate to Severe | 56.30 | 76.11 |  |  |  |  |  |
| Yang 2017 | Control | Community | Dementia | Moderate to Severe | 54.30 | 74.67 |  |  |  |  |  |
| Yang 2021 | Control | Nursing home | Alzheimer’s Disease | Severe | 81.97 | 85.64 |  |  |  | 75 | 18.67 |
| Yang 2021 | Intervention | Nursing home | Alzheimer’s Disease | Severe | 70.77 | 85.11 |  |  |  | 75 | 13.33 |
| Zarepour 2020 | Control | Hospital admission | Dementia | Not specified |  | 73.60 | 71.40 | 46.80 |  | 35 | 0.00 |
| Zarepour 2020 | Intervention | Hospital admission | Dementia | Not specified |  | 73.60 | 68.60 | 46.80 |  | 35 | 0.00 |
| Zhang 2021 | Intervention | Nursing home | Alzheimer’s Disease | Moderate to Severe | 70.59 | 76.80 |  | 34.02 |  |  |  |
| Zhang 2021 | Control | Nursing home | Alzheimer’s Disease | Moderate to Severe | 76.09 | 76.41 |  | 33.47 |  |  |  |
| ZHAO 2010 | Control | Hospital admission | Dementia | Not specified | 60.00 | 68.41 |  |  |  |  |  |
| ZHAO 2010 | Intervention | Hospital admission | Dementia | Not specified | 60.00 | 68.41 |  |  |  |  |  |

1. Risk of bias assessments of individual studies

1. Egger’s test results

|  | **k** | **Intercept** | **Estimate** | **SE** | **t** | **p** |
| --- | --- | --- | --- | --- | --- | --- |
| **Caregiver outcomes** |  |  |  |  |  |  |
| perceived burden | 18 | -2.9269 | 3.6619 | 1.4881 | 2.46 | 0.0256 |
| **Care recipient outcomes** |  |  |  |  |  |  |
| neuropsychiatric symptoms | 11 | -0.0811 | -1.2912 | 0.8651 | -1.49 | 0.1697 |
| cognitive function | 10 | 0.8391 | -0.5108 | 1.8810 | -0.27 | 0.7928 |

1. Funnel plots
   1. Perceived burden of carers^[[3]](#footnote-3)^

- 1. Neuropsychiatric symptoms of care recipients^[[4]](#footnote-4)^

- 1. Cognitive function of care recipients^[[5]](#footnote-5)^

1. Additional forest plots
   1. Care-related distress

- 1. Carer quality of life

- 1. Carer anxiety

- 1. Neuropsychiatric symptoms of care recipients

- 1. Cognitive function of care recipients

- 1. Quality of life of care recipients

- 1. Composite activities of daily life of care recipients

1. Leave-one-out analysis
   1. Care-related distress

- 1. Perceived burden of carers

- 1. Carer depression

- 1. Carers’ quality of life

- 1. Carer anxiety

- 1. Cognitive function of care recipients

- 1. Neuropsychiatric symptoms of care recipients

- 1. Quality of life of care recipients

- 1. Composite activities of daily life of care recipients

1. Summary of subgroup analyses for pre-determined factors

| Subgroup | k | TE | 95% CI | p | I^2^ | 95% CI | P _subgroup_ |
| --- | --- | --- | --- | --- | --- | --- | --- |
| Intervention type | |  |  |  |  |  | 0.1129 |
| Training for dementia and self care | 6 | -0.8889 | [-1.3958, -0.3821] | 0.0006 | 0.3066 | [0.0000, 0.7180] |  |
| Training for BPSD management | 4 | -0.5939 | [-1.0625, -0.1254] | 0.013 | 0 | [0.0000, 0.8469] |  |
| Comprehensive care for dementia patients | 2 | -1.1995 | [-1.8599, -0.5392] | 0.0004 | 0.3043 | NA |  |
| Guided self-study | 1 | -0.2261 | [-1.2859, 0.8337] | 0.6758 | NA | NA |  |
| Music therapy for dementia patients | 1 | -0.8765 | [-1.9028, 0.1497] | 0.0941 | NA | NA |  |
| Spiritual group therapy | 1 | -2.2900 | [-3.4117, -1.1682] | 0.0001 | NA | NA |  |
| Cognitive and functional training for dementia patients | 1 | -1.3419 | [-2.2020, -0.4818] | 0.0022 | NA | NA |  |
| Aromatherapy | 1 | -0.1963 | [-1.7921, 1.3994] | 0.8094 | NA | NA |  |
| Source of participants | |  |  |  |  |  | 0.0042 |
| Hospital admission | 7 | -0.6284 | [-1.0006, -0.2562] | 0.0009 | 0 | [0.0000, 0.7081] |  |
| Community | 6 | -0.8014 | [-1.2993, -0.3035] | 0.0016 | 0.1245 | [0.0000, 0.7778] |  |
| Patient registration | 2 | -2.3363 | [-3.2040, -1.4686] | 0 | 0 | NA |  |
| Nursing home | 2 | -1.0831 | [-1.6032, -0.5629] | 0 | 0 | NA |  |
| Severity of dementia | |  |  |  |  |  | 0.9427 |
| Not specified | 7 | -0.9560 | [-1.6779, -0.2341] | 0.0094 | 0.5847 | [0.0413, 0.8201] |  |
| Any severity stage | 4 | -0.9320 | [-1.4430, -0.4210] | 0.0004 | 0.2574 | [0.0000, 0.7162] |  |
| Mild to moderate | 4 | -0.9345 | [-1.4343, -0.4347] | 0.0002 | 0 | [0.0000, 0.8469] |  |
| Mild | 1 | -0.4883 | [-1.4522, 0.4755] | 0.3207 | NA | NA |  |
| Severe | 1 | -0.9339 | [-1.5870, -0.2808] | 0.0051 | NA | NA |  |
| Intervention frequency |  |  |  |  |  |  | 0.2093 |
| Weekly | 8 | -1.2290 | [-1.6817, -0.7764] | 0 | 0.384 | [0.0000, 0.7280] |  |
| Monthly to weekly | 6 | -0.7331 | [-1.1094, -0.3568] | 0.0001 | 0 | [0.0000, 0.7462] |  |
| > Weekly | 2 | -0.6775 | [-1.5407, 0.1857] | 0.124 | 0 | NA |  |
| N/A | 1 | -0.2261 | [-1.2859, 0.8337] | 0.6758 | NA | NA |  |
| Funding type | |  |  |  |  |  | 0.8279 |
| Academic | 5 | -1.1310 | [-1.8154, -0.4466] | 0.0012 | 0.5447 | [0.0000, 0.8321] |  |
| International | 4 | -0.6278 | [-1.1663, -0.0893] | 0.0223 | 0 | [0.0000, 0.8469] |  |
| No information | 4 | -1.0541 | [-2.0162, -0.0920] | 0.0318 | 0.6055 | [0.0000, 0.8681] |  |
| Self-fund | 2 | -0.8268 | [-1.3895, -0.2641] | 0.004 | 0 | NA |  |
| Local | 2 | -0.8103 | [-1.4053, -0.2153] | 0.0076 | 0 | NA |  |
| Country |  |  |  |  |  |  | 0.6790 |
| Turkey | 6 | -0.7754 | [-1.1816, -0.3691] | 0.0002 | 0.3207 | [0.0000, 0.7254] |  |
| India | 2 | -0.3045 | [-1.0447, 0.4358] | 0.4202 | 0 | NA |  |
| China | 2 | -1.1995 | [-1.8599, -0.5392] | 0.0004 | 0.3043 | NA |  |
| Iran | 2 | -1.3407 | [-3.2072, 0.5258] | 0.1592 | 0.8162 | [0.2209, 0.9566] |  |
| Peru | 1 | -1.0353 | [-2.1307, 0.0600] | 0.0639 | NA | NA |  |
| Vietnam | 1 | -0.9447 | [-2.0698, 0.1804] | 0.0998 | NA | NA |  |
| Brazil | 1 | -0.2075 | [-1.6500, 1.2351] | 0.778 | NA | NA |  |
| Argentina | 1 | -1.3419 | [-2.2020, -0.4818] | 0.0022 | NA | NA |  |
| Egypt | 1 | -0.8916 | [-1.9303, 0.1472] | 0.0925 | NA | NA |  |

1. Summary of meta-regression for pre-determined factors

|  | (1) | (2) | (3) | (4) | (5) | (6) | (7) | (8) | (9) |
| --- | --- | --- | --- | --- | --- | --- | --- | --- | --- |
| intercept | 17.001 | -0.335 | -2.486* | -1.609 | -0.859 | -1.084*** | -1.099*** | -0.795* | -5.349* |
|  | (62.956) | (1.260) | (1.172) | (2.104) | (0.550) | (0.218) | (0.260) | (0.311) | (2.261) |
| Year | -0.009 |  |  |  |  |  |  |  |  |
|  | (0.031) |  |  |  |  |  |  |  |  |
| Mean age of caregivers |  | -0.010 |  |  |  |  |  |  |  |
|  |  | (0.023) |  |  |  |  |  |  |  |
| Female percent of caregivers |  |  | 0.021 |  |  |  |  |  |  |
|  |  |  | (0.015) |  |  |  |  |  |  |
| Mean age of dementia patients |  |  |  | 0.010 |  |  |  |  |  |
|  |  |  |  | (0.027) |  |  |  |  |  |
| Female percent of dementia patients |  |  |  |  | 0.001 |  |  |  |  |
|  |  |  |  |  | (0.009) |  |  |  |  |
| Attrition rate |  |  |  |  |  | 0.017 |  |  |  |
|  |  |  |  |  |  | (0.011) |  |  |  |
| Treatment duration |  |  |  |  |  |  | 0.003 |  |  |
|  |  |  |  |  |  |  | (0.003) |  |  |
| Sample size |  |  |  |  |  |  |  | -0.001 |  |
|  |  |  |  |  |  |  |  | (0.004) |  |
| Regional HDI |  |  |  |  |  |  |  |  | 5.437+ |
|  |  |  |  |  |  |  |  |  | (2.790) |
| Num.Obs. | 15 | 12 | 12 | 14 | 14 | 10 | 15 | 15 | 11 |
| AIC | 35.0 | 31.8 | 25.6 | 31.9 | 27.8 | 23.6 | 34.2 | 35.3 | 26.5 |
| BIC | 37.1 | 33.2 | 27.1 | 33.8 | 29.8 | 24.6 | 36.3 | 37.4 | 27.7 |
| i2 | 0.113 | 0.330 | 0 | < 0.001 | < 0.001 | 0.076 | 0.148 | 0.148 | < 0.001 |
| + p < 0.1, * p < 0.05, ** p < 0.01, *** p < 0.001 | | | | | | | | |  |

1. Bubble plots of meta-regression for caregiver burden


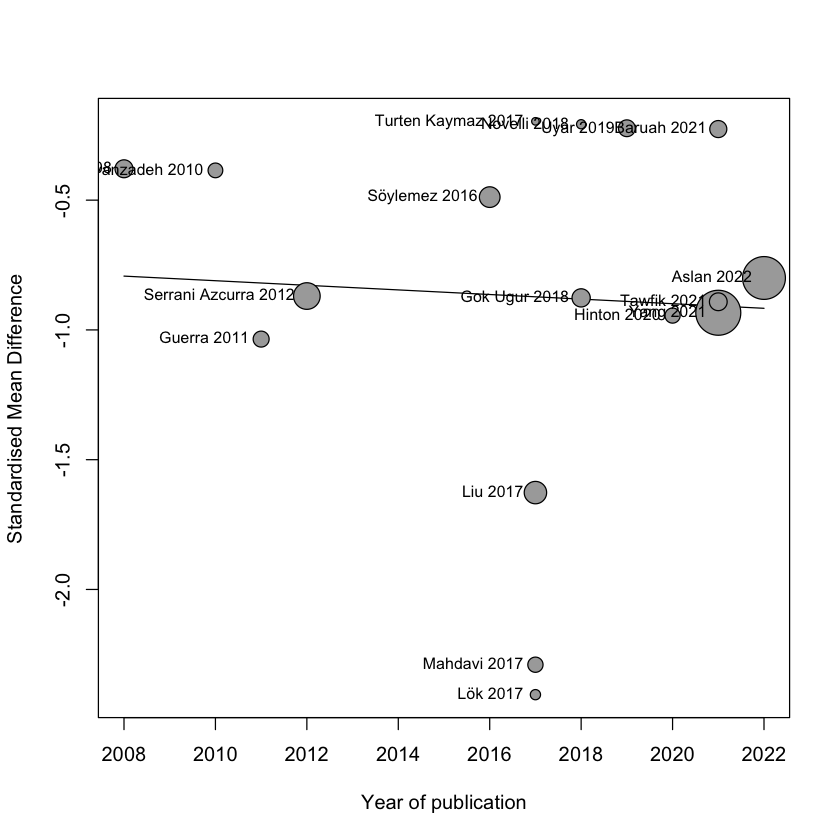

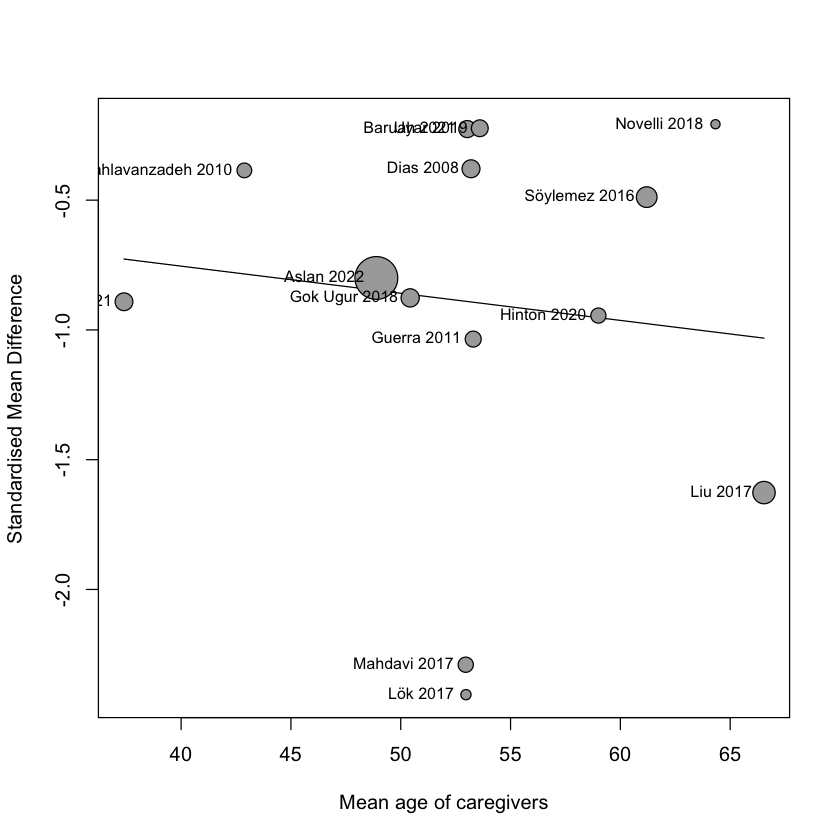

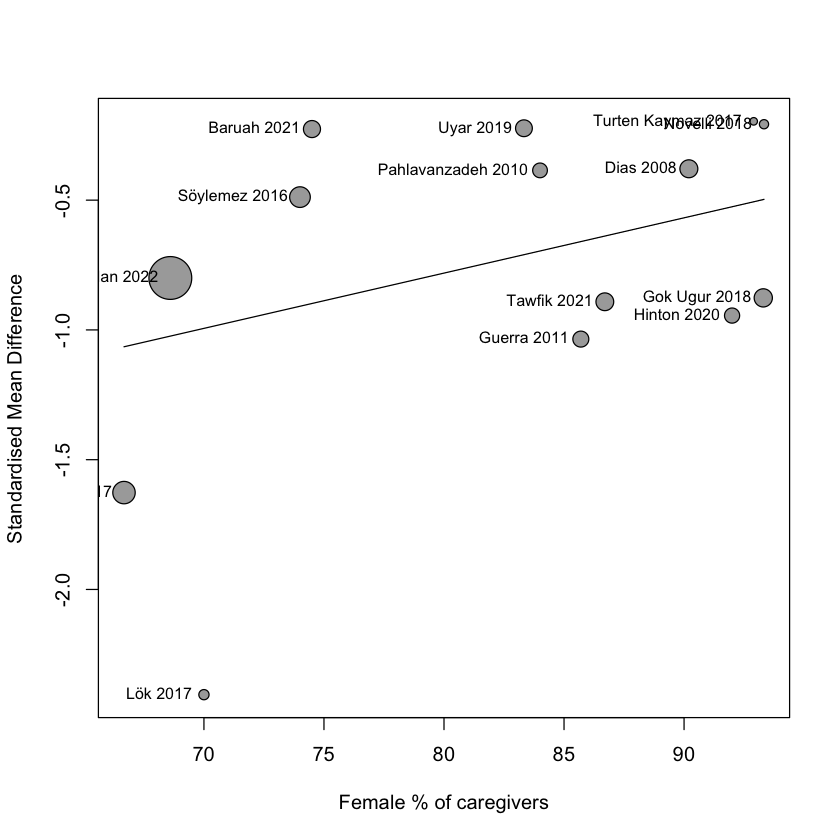

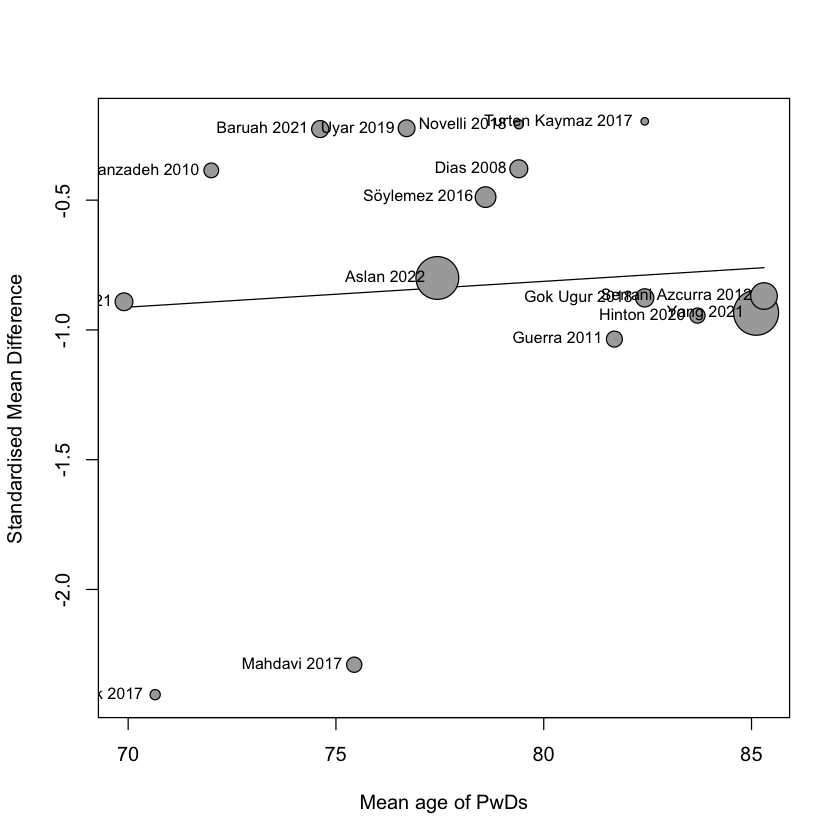

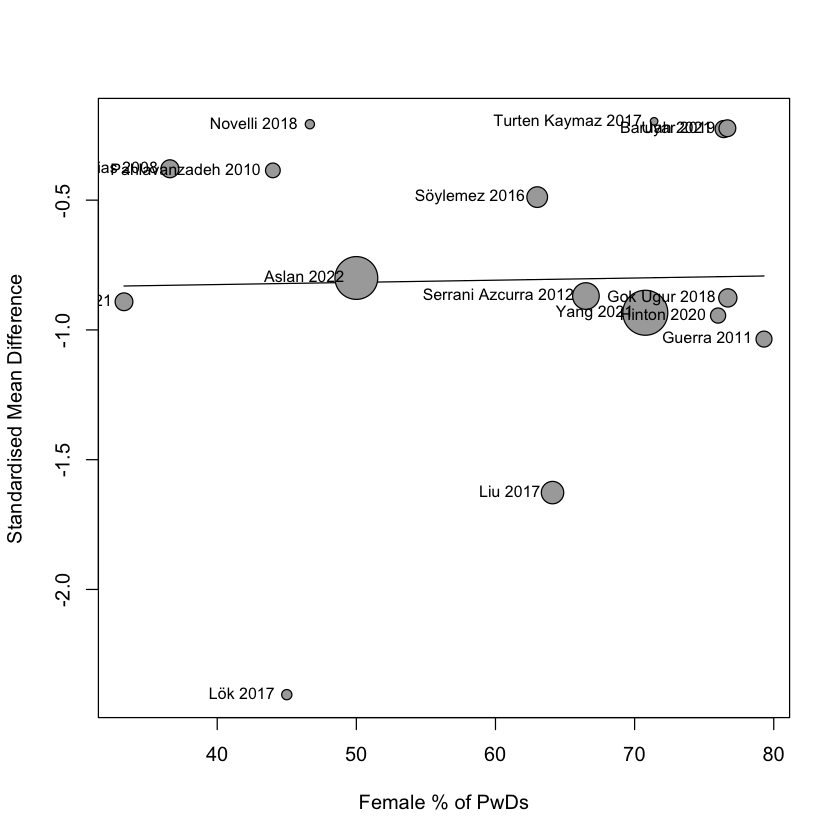

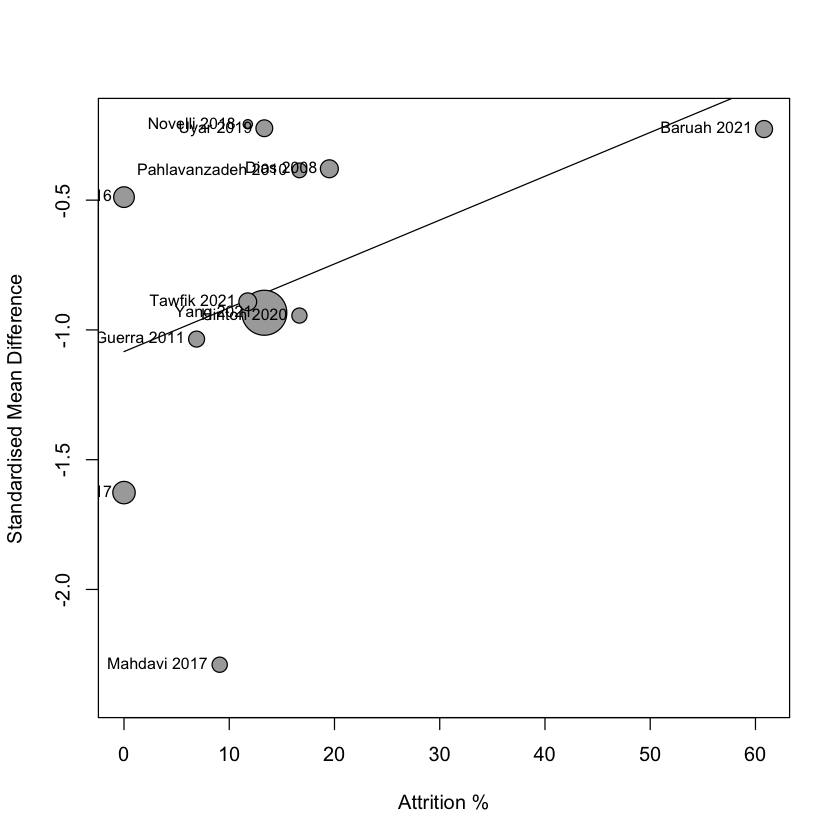

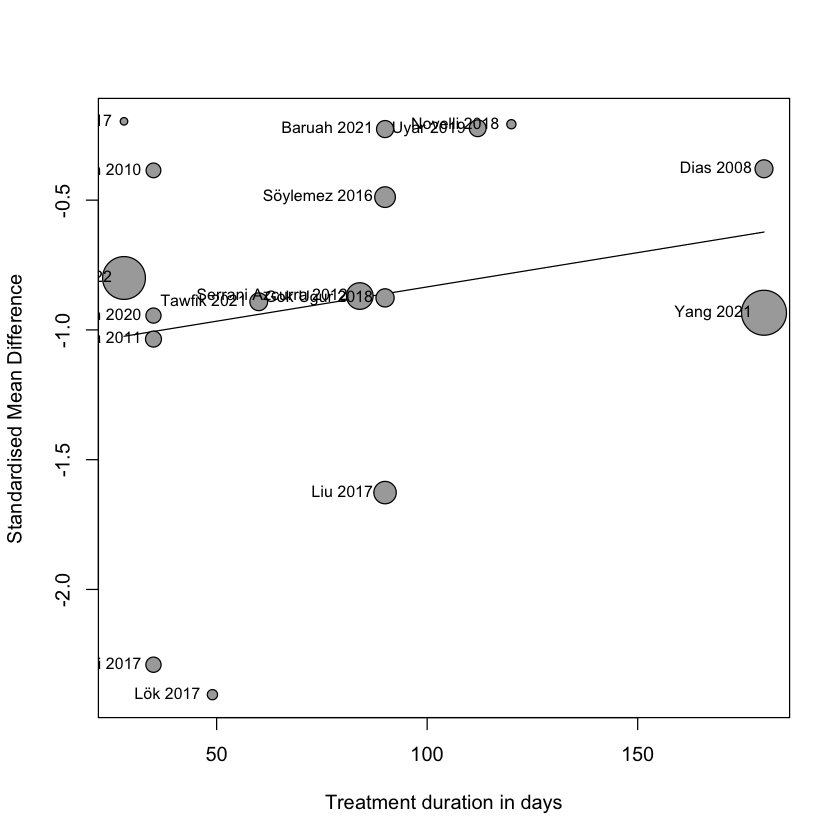

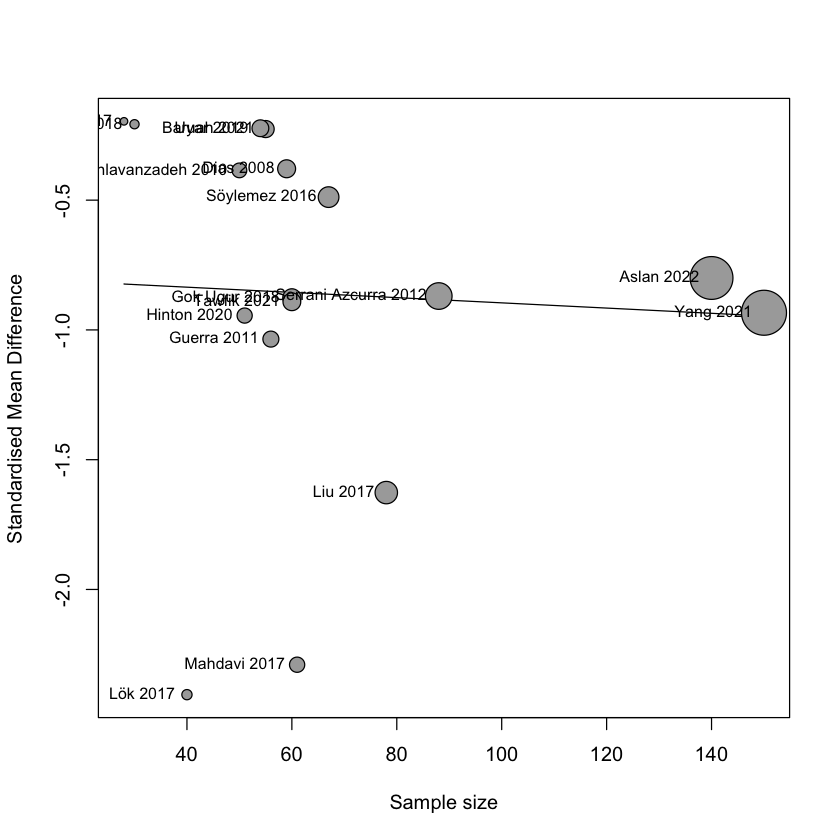

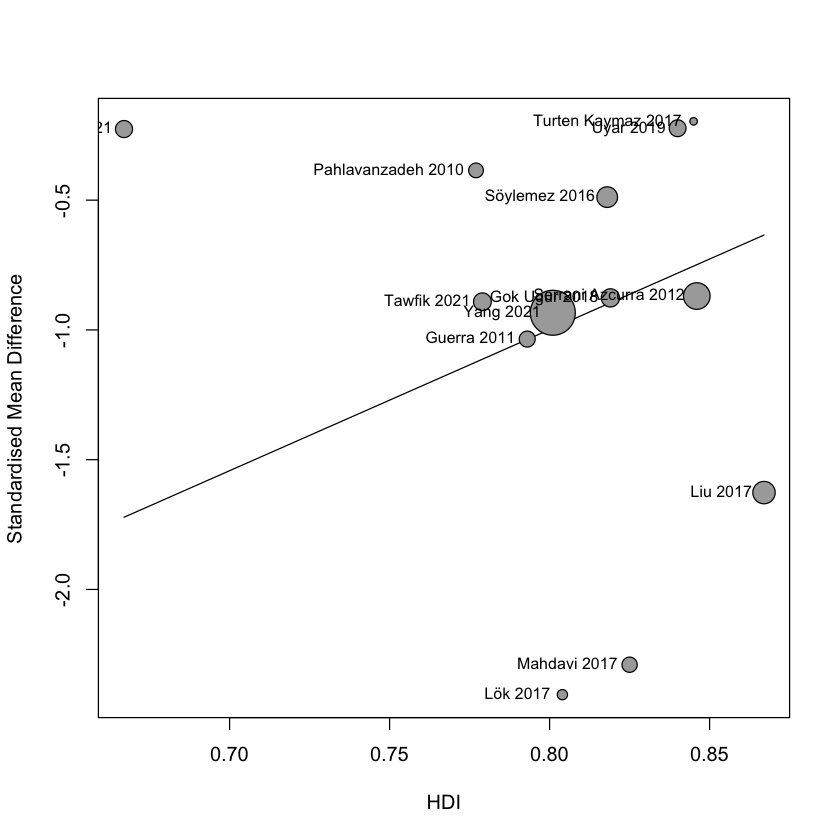


1. MetaForest results
   1. Variable importance plot

- 1. Partial dependency plot

1. Citations of included studies

| Source | Study Identifier | Formatted citation |
| --- | --- | --- |
| STRiDE review | Dias 2008 | Dias, Amit; Dewey, M. E.; D'Souza, J.; Rajesh, Dhume; Motghare, D. D.; Shaji, K. S.; Menon, R.; Prince, M.; Vikram, Patel. The effectiveness of a home care program for supporting caregivers of persons with dementia in developing countries: a randomised controlled trial from Goa, India. PLos One 2008;():e2333. |
| STRiDE review | Gok Ugur 2018 | Gok Ugur, Hacer; Orak, Oya Sevcan; Yaman Aktas, Yesim; Enginyurt, Ozgur; Saglambilen, Okan. Effects of Music Therapy on the Care Burden of In-Home Caregivers and Physiological Parameters of Their In-Home Dementia Patients: A Randomized Controlled Trial Complementary medicine research. 2018. |
| STRiDE review | Guerra 2011 | Guerra, M.; Ferri, C. P.; Fonseca, M.; Banerjee, S.; Prince, M. Helping carers to care: the 10/66 dementia research group's randomized control trial of a caregiver intervention in Peru. Revista brasileira de psiquiatria (são paulo, brazil : 1999) 2011;33(1):47‐54 |
| STRiDE review | He 2012 | He, L.-C. The research of 3R thearpy improving cognitive of patients with Alzheimer’s disease. China Medicine [中国医药] 2012;7(6):693-695 |
| STRiDE review | JIANG 2012 | JIANG, Gaoxuan; LI, Mingqiu; HUANG, Haihua; CHAI, Zhenfang; WU, Ailian; ZHANG, Guosheng. The curative effect of whole course rehabilitation nursing intervention on mild-to-moderate Alzheimer's disease patients [全程康复护理干预对阿尔茨海默病的疗效观察]. China Practical Medical 2012;7(8):16-18 |
| STRiDE review | Kamkhagi 2015 | Kamkhagi, D.; Costa, A. C. O.; Kusminsky, S.; Supino, D.; Diniz, B. S.; Gattaz, W. F.; Forlenza, O. V. Benefits of psychodynamic group therapy on depression, burden and quality of life of family caregivers to Alzheimer's disease patients. Revista de psiquiatria clinica 2015-1-1 2015;42(6):157‐160 |
| STRiDE review | Liu 2017 | Liu, X.; Chen, L.; Jiang, C. Influence of family visit of community nurses for the elderly with dementia family caregivers' burden of care and quality of life of the elderly with dementia. Chinese nursing research 2017-1-1 2017;31(33):4206‐4209 |
| STRiDE review | Lök 2017 | Lök, N.; Bademli, K. Pilot testing of the "First You Should Get Stronger" program among caregivers of older adults with dementia. Archives of gerontology and geriatrics 2017;68():84‐89 |
| STRiDE review | Lök 2019 | Lök, Neslihan; Bademli, Kerime; Selcuk-Tosun, Alime. The effect of reminiscence therapy on cognitive functions, depression, and quality of life in Alzheimer patients: Randomized controlled trial. International Journal of Geriatric Psychiatry 2019-1-1 2019;34(1):47-53 |
| STRiDE review | Mahdavi 2017 | Mahdavi, Behrouz; Fallahi-Khoshknab, Masoud; Mohammadi, Farahnaz; Hosseini, Mohammad Ali; Haghi, Marjan. Effects of spiritual group therapy on caregiver strain in home caregivers of the elderly with Alzheimer's disease. Archives of psychiatric nursing 2017-1-1 2017;31(3):269-273 |
| STRiDE review | Norouzi 2014^[[6]](#footnote-6)^ | Norouzi, Mercedeh; Mahmood, Golzari; Faramarz, Sohrabi. Effectiveness of mindfulness based cognitive therapy on the quality of life, depression and burden of demented women caregivers. Zahedan J. Res. Med. Sci. 2014-1-1 2014;16(9):5-11 |
| STRiDE review | Novelli 2018 | Novelli, Marcia M. P. C.; Machado, Styfany C. B.; Lima, Gabriela B.; Cantatore, Lais; Sena, Barbara P.; Rodrigues, Renata S.; Rodrigues, Camila I. B.; Canon, Mariana B. F.; Piersol, Catherine V.; Nitrini, Ricardo; Yassuda, Monica S.; Gitlin, Laura N. Effects of the Tailored Activity Program in Brazil (TAP-BR) for Persons With Dementia: A Randomized Pilot Trial. Alzheimer disease and associated disorders 2018;32(4):339-345. |
| STRiDE review | Pahlavanzadeh 2010 | Pahlavanzadeh, Saeed; Heidari, Fatemeh Ghaedi; Maghsudi, Jahangir; Ghazavi, Zahra; Samari, Saeed. The effects of family education program on the caregiver burden of families of elderly with dementia disorders. Iranian journal of nursing and midwifery research 2010;15(3):102-8 |
| STRiDE review | Pankong 2018 | Pankong, O.; Linchong, P.; Khanokporn, S.; Totsaporn, K. A randomized controlled trial of enhancing positive aspects of caregiving in Thai dementia caregivers for dementia. Pacific rim international journal of nursing research 2018-1-1 2018;22(2):131‐133 |
| STRiDE review | Salamizadeh 2017^[[7]](#footnote-7)^ | Salamizadeh, Azam Ms Student; Mirzaei, Tayebeh PhD; Ravari, Ali PhD. The Impact of Spiritual Care Education on the Self-Efficacy of the Family Caregivers of Elderly People with Alzheimer's Disease. International journal of community based nursing and midwifery 2017-1-1 2017;5(3):231-238 |
| STRiDE review | Serrani Azcurra 2012 | Serrani Azcurra, D. J. A reminiscence program intervention to improve the quality of life of long-term care residents with Alzheimer's disease: a randomized controlled trial. Revista brasileira de psiquiatria (sao paulo, brazil : 1999) 2012;34(4):422‐433 |
| STRiDE review | Shata 2017 | Shata, Zeinab Nazeeh; Amin, Marwa R.; El-Kady, Heba M.; Abu-Nazel, Mervat W. Efficacy of a multi-component psychosocial intervention program for caregivers of persons living with neurocognitive disorders, Alexandria, Egypt: A randomized controlled trial. Avicenna journal of medicine 2017;7(2):54-63. |
| STRiDE review | Söylemez 2016 | Söylemez, Burcu Akpinar; Küçükgüçlü, Özlem; Buckwalter, Kathleen C. Application of the Progressively Lowered Stress Threshold Model with Community-Based Caregivers. Journal of Gerontological Nursing 2016;42(7):44-54 |
| STRiDE review | SU 2012 | SU, Xiaoling. Application effect of comprehensive nursing intervention in patients with Alzheimer's disease [综合护理干预在老年痴呆患者中的应用效果]. Today Nurse 2012;3():116-118 |
| STRiDE review | SUN 2010 | SUN, Xiaohui; SU, Haidan; LI, Xuejie. Influence of nursing intervention on quality of life of patients with Alzheimer disease [护理干预对阿尔茨海默病病人生活质量的影响]. Chinese Nursing Research 2010;24(13):1167-1169 |
| STRiDE review | TAN 2010 | TAN, Xiaoxue; LIN, Chunjie; ZHAO, Liwen; CHEN, Rulan; YANG, Bingyu; HUANG, Julan; LIU, Hongrui. Effects of Early Family Nursing Intervention on Quality of Life of Patients with Alzheimer's Disease [早期家庭护理干预对阿尔茨海默病患者生活质量的影响]. Nursing Journal of Chinese People's Liberation Army [解放军护理杂志] 2010;27(3):176-179. |
| STRiDE review | Wang 2010 | Wang, Zhou; Mu, JiangBing; Liu, Rui. Community nursing intervention on quality of life in patients with senile dementia. Modern Preventive Medicine 2010;37(5):840-841, 844 |
| STRiDE review | Wang 2012 | Wang, Li-Qun; Chien, Wai-Tong; Lee, Isabella Y. M. An experimental study on the effectiveness of a mutual support group for family caregivers of a relative with dementia in mainland China. Contemporary nurse 2012;40(2):210-224 |
| STRiDE review | Wang 2014 | Wang, XiaoXiang; Zhang, Ru; Liu, WenQing. Effects of home care intervention on the rehabilitation of patients with Alzheimer's disease. Medical Journal of National Defending Forces in Southwest China 2014;24(1):85-86 |
| STRiDE review | Wang 2017c | Wang, F.; Xiao, L. D.; Wang, K.; Li, M.; Yang, Y. Evaluation of a WeChat-based dementia-specific training program for nurses in primary care settings: a randomized controlled trial. Applied nursing research 2017;38():51‐59 |
| STRiDE review | Wang 2017e | Wang, Y.; Xiao, L. D.; Ullah, S.; He, G. P.; De Bellis, A. Evaluation of a nurse-led dementia education and knowledge translation programme in primary care: a cluster randomized controlled trial. Nurse education today 2017;49():1‐7 |
| STRiDE review | Yang 2017 | Yang, D. L.; Li, X.; Su, N.; Ji, W. D.; Chen, X. X. Family medical intervention model of senile dementia with behavioral and psychological symptoms. Journal of shanghai jiaotong university (medical science) 2017;37(3):398‐402 |
| STRiDE review | ZHAO 2010 | ZHAO, Liao; TAN, Xiaoxue; CHEN, Shaomei. Effect of home nursing intervention on patients with Alzheimer's disease [对阿尔茨海默病患者实施家庭护理干预的效果观察]. Journal of Qiqihar University of Medicine 2010;31(6):948-949 |
| 2022 search | Chen 2020 | Chen M, Bai Y, Zhou X, Chen W, He D, Li Y. Effect of Extended Nursing on the Behavioral and Psychological Symptoms and Cognitive Dysfunction of Patients with Moderate and Severe Alzheimer’s Disease. Indian J Pharm Sci. 2020;82. doi:10.36468/pharmaceutical-sciences.spl.137 |
| 2022 search | Ghaffari 2019 | Ghaffari F, Rostami M, Fotokian Z, Hajiahmadi M. Effectiveness of Resilience Education in the Mental Health of Family Caregivers of Elderly Patients with Alzheimer’s Disease. Iran J Psychiatry Behav Sci. 2019;13(3). doi:10.5812/ijpbs.69507 |
| 2022 search | Govindakumari 2020 | Govindakumari R, Goothy SSK, Raghvan V. Effectiveness of home-based cognitive training program on a cognitive level, the activity of daily living among elderly persons with mild cognitive impairment in selected community settings of Puducherry. International Journal of Research in Pharmaceutical Sciences. 2020;11(4):5301-5309. doi:10.26452/ijrps.v11i4.3149 |
| 2022 search | Hinton 2020 | Hinton L, Nguyen H, Nguyen HT, et al. Advancing family dementia caregiver interventions in low‐ and middle‐income countries: A pilot cluster randomized controlled trial of Resources for Advancing Alzheimer’s Caregiver Health in Vietnam (REACH VN). A&D Transl Res & Clin Interv. 2020;6(1). doi:10.1002/trc2.12063 |
| 2022 search | Pan 2019 | Pan Y, Chen R, Yang D. The Role of Mutuality and Coping in a Nurse-Led Cognitive Behavioral Intervention on Depressive Symptoms Among Dementia Caregivers. Research in Gerontological Nursing. 2019;12(1):44-55. doi:10.3928/19404921-20181212-01 |
| 2022 search | Uyar 2019 | Uyar F, Özmen D, Mavioğlu H, Atalay N. Assessment of the impact of dementia care and support program in both patient and caregiver outcomes: an intervention study. Türk Geriatri Dergisi. 2019;22(4):482-493. doi:10.31086/tjgeri.2020.127 |
| 2022 search | Zarepour 2020 | Zarepour A, Hazrati M, Kadivar AA. The Impact of Educational Intervention on the Anxiety of Family Caregivers of the Elderly with Dementia: A Randomized Controlled Tri. IJCBNM. 2020;8(3). doi:10.30476/ijcbnm.2020.81680.0 |
| 2023 search | Aslan 2022 | Aslan S, Aylaz R. The Effect of the Progressively Lowered Stress Threshold Model Training Program on Depression and Care Burden in Dementia Patient Caregivers. Galician med j. 2022;29(2):E202223. doi:10.21802/gmj.2022.2.3 |
| 2023 search | Baruah 2021 | Baruah U, Varghese M, Loganathan S, et al. Feasibility and preliminary effectiveness of an online training and support program for caregivers of people with dementia in India: a randomized controlled trial. Int J Geriatr Psychiatry. 2021;36(4):606-617. doi:10.1002/gps.5502 |
| 2023 search | Duru Asiret 2021 | Duru Aşiret G, Kütmeç Yılmaz C, Sayın Kasar K. Investigation of the effects of interventions made according to the P rogressively L owered S tress T hreshold M odel on the care outcomes of A lzheimer patients and their families: a randomized clinical trial. Psychogeriatrics. 2021;21(5):738-748. doi:10.1111/psyg.12734 |
| 2023 search | Jahani 2022 | Jahani L, Abolhassani S, Babaee S, Omranifard V. Effects of a compassion-based program on the grief experienced by caregivers of people suffering from dementia: a randomized controlled clinical trial. BMC Nurs. 2022;21(1):198. doi:10.1186/s12912-022-00980-5 |
| 2023 search | Tawfik 2021 | Tawfik NM, Sabry NA, Darwish H, Mowafy M, Soliman SSA. Psychoeducational Program for the Family Member Caregivers of People with Dementia to Reduce Perceived Burden and Increase Patient’s Quality of Life: A Randomized Controlled Trial. J Prim Care Community Health. 2021;12:215013272110140. doi:10.1177/21501327211014088 |
| 2023 search | Wang 2021 | Wang S, Cheung DSK, Leung AYM, Davidson PM. Bibliotherapy for improving caregiving appraisal of informal caregivers of people with dementia: A pilot randomized controlled trial. Res Nurs Health. 2021;44(4):692-703. doi:10.1002/nur.22143 |
| 2023 search | Xu 2022 | Xu XY, Kwan RYC, Miao J, Chai A, Leung AYM. Telephone-Based Behavioral Activation for Improving Sleep Quality in Family Caregivers of People With Dementia: A Pilot Randomized Controlled Trial. Behavior Therapy. 2022;53(5):887-899. doi:10.1016/j.beth.2022.02.007 |
| 2023 search | Yang 2021 | Yang L, Xuan C, Yu C, Jin X, Zheng P, Yan J. Effects of comprehensive intervention on life quality among the elderly with Alzheimer Disease and their caregivers based on mixed models. Nursing Open. 2022;9(2):1412-1422. doi:10.1002/nop2.917 |
| 2023 search | Zhang 2021 | Zhang J, Wang Z, Li Y, et al. Effects of a Caregiver Training Program on Oral Hygiene of Alzheimer’s Patients in Institutional Care. Journal of the American Medical Directors Association. 2021;22(7):1429-1434.edoi:10.1016/j.jamda.2020.12.042 |
| other reviews | Arango-Lasprilla 2014 | Arango-Lasprilla JC, Panyavin I, Merchán EJH, et al. Evaluation of a Group Cognitive–Behavioral Dementia Caregiver Intervention in Latin America. Am J Alzheimers Dis Other Demen. 2014;29(6):548-555. doi:10.1177/1533317514523668 |
| other reviews | Danucalov 2013 | Danucalov MAD, Kozasa EH, Ribas KT, et al. A Yoga and Compassion Meditation Program Reduces Stress in Familial Caregivers of Alzheimer’s Disease Patients. Evidence-Based Complementary and Alternative Medicine. 2013;2013:1-8. doi:10.1155/2013/513149 |
| other reviews | Heydari 2017 | Heidari S, Mirzaei T, Heydarinezad Chatrodi M, Heidarzadeh A. Effect of problem-oriented coping strategies training on perceived stress in the family caregivers of the elderly with Alzheimer. Hayat. 2020;26(4):384-395. Accessed December 25, 2022. http://hayat.tums.ac.ir/article-1-3818-en.html |
| other reviews | Oliveira 2018 | de Oliveira AM, Radanovic M, Homem de Mello PC, et al. An intervention to reduce neuropsychiatric symptoms and caregiver burden in dementia: Preliminary results from a randomized trial of the tailored activity program-outpatient version. Int J Geriatr Psychiatry. 2019;34(9):1301-1307. doi:10.1002/gps.4958 |
| other reviews | Oliveira 2021 | Oliveira AM, Radanovic M, Mello PCH de, et al. Adjunctive Therapy to Manage Neuropsychiatric Symptoms in Moderate and Severe Dementia: Randomized Clinical Trial Using an Outpatient Version of Tailored Activity Program. JAD. 2021;83(1):475-486. doi:10.3233/JAD-210142 |
| other reviews | Turten Kaymaz 2017 | Turten Kaymaz T, Ozdemir L. Effects of aromatherapy on agitation and related caregiver burden in patients with moderate to severe dementia: A pilot study. Geriatric Nursing. 2017;38(3):231-237. doi:10.1016/j.gerinurse.2016.11.001 |
| other reviews | Villareal-Reyna 2012^[[8]](#footnote-8)^ | Villareal-Reyna M de los Á, Salazar-González BC, Cruz-Quevedo JE, Carrillo-Cervantes AL, Champion JD. Outcomes of Interventions for Alzheimer’s Family Caregivers in Mexico. West J Nurs Res. 2012;34(7):973-990. doi:10.1177/0193945910387283 |

1. List of collaborators in the STRiDE Evidence Review Group

| Name | Affiliation |
| --- | --- |
| Suvarna Alladi | National Institute of Mental Health and Neuro Sciences, Bangalore, India |
| Claudia Iveth Astudillo-García | National Institute of Psychiatry "Ramón de la Fuente Muñiz", Epidemiological and Psychosocial Research Directorate. Mexico City, Mexico |
| Kirsten Bobrow | (1) Division of Endocrinology, Department of Medicine, University of Cape Town, Cape Town, South Africa; (2) The Global Brain Health Institute, the University of California at San Francisco, United States |
| Xinxin Cai | Department of Social Work and Social Administration, The University of Hong Kong, Hong Kong |
| ShanShan Cheng | Department of Social Work and Social Administration, The University of Hong Kong, Hong Kong |
| Jacky Choy | Department of Social Work and Social Administration, The University of Hong Kong, Hong Kong |
| Adelina Comas-Herrera | Care Policy and Evaluation Centre (CPEC), Department of Health Policy, London School of Economics and Political Science, United Kingdom |
| Fabiana A. F. Da Mata | Department of Psychiatry, School of Medicine , Universidade Federal de São Paulo (UNIFESP), São Paulo, Brazil |
| Ruizhi Dai | (1) Faculty of Social Sciences, The University of Hong Kong, Hong Kong; (2) Department of Psychology, Faculty of Education, Guangzhou University, Guangzhou, China |
| Andra Fry | Library, London School of Economics and Political Science, United Kingdom |
| Saadiya Hurzuk | National Institute of Mental Health and Neuro Sciences, Bangalore, India |
| Martin Knapp | Care Policy and Evaluation Centre (CPEC), Department of Health Policy, London School of Economics and Political Science, United Kingdom |
| Monisha Lakshminarayanan | Schizophrenia Research Foundation (SCARF), Chennai, India |
| Dara Kiu Yi Leung | Department of Social Work and Social Administration, The University of Hong Kong, Hong Kong |
| Mariana Lopez-Ortega | National Institute of Geriatrics, National Institutes of Health, Mexico City, Mexico |
| Klara Lorenz-Dant | Care Policy and Evaluation Centre (CPEC), Department of Health Policy, London School of Economics and Political Science, United Kingdom |
| David McDaid | Care Policy and Evaluation Centre (CPEC), Department of Health Policy, London School of Economics and Political Science, United Kingdom |
| Christine Musyimi | Africa Mental Health Research and Training Foundation, Nairobi, Kenya |
| Huseyin Naci | Department of Health Policy, London School of Economics and Political Science, United Kingdom |
| Gayathri Nagarajan | Schizophrenia Research Foundation (SCARF), Chennai, India |
| David Ndetei | (1) University of Nairobi; (2) Africa Mental Health Research and Training Foundation (AMHRTF), Nairobi, Kenya; (3) World Psychiatric Association Collaborating Centre for Research and Training, AMHRTF, Nairobi, Kenya |
| Tuan Anh Nguyen | (1) Division of Social Gerontology, National Ageing Research Institute, Melbourne, Australia; (2) School of Health Sciences, Swinburne University of Technology, Melbourne, Australia; (3) UniSA Clinical & Health Sciences, University of South Australia, Adelaide, Australia; (4) Health Strategy and Policy Institute, Ministry of Health of Vietnam, Hanoi, Vietnam |
| Deborah Oliveira | (1) Universidad Andrés Bello, Faculty of Nursing, Campus Viña del Mar, Chile; (2) Millennium Institute for Care Research (MICARE), Santiago, Chile; (3) Universidade Federal de São Paulo, School of Medicine, Department of Psychiatry, São Paulo, Brazils |
| Disha Patel | Care Policy and Evaluation Centre (CPEC), Department of Health Policy, London School of Economics and Political Science, United Kingdom |
| Aditya Putra | Atma Jaya Catholic University of Indonesia, Jakarta, Indonesia |
| Harleen Rai | University of Nottingham, Nottingham, United Kingdom |
| Jayeeta Rajagopalan | National Institute of Mental Health and Neuro Sciences, Bangalore, India |
| Maximilian Salcher-Konrad | (1) Care Policy and Evaluation Centre (CPEC), Department of Health Policy, London School of Economics and Political Science, United Kingdom; (2) WHO Collaborating Centre for Pharmaceutical Pricing and Reimbursement Policies, Pharmacoeconomics Department, Austrian National Public Health Institute (Gesundheit Österreich / GÖG), Vienna, Austria |
| Tara Sani | Alzheimer’s Indonesia, Indonesia |
| Cheng Shi | (1) School of Graduate Studies & Institute of Policy Studies, Lingnan University, Hong Kong; (2) Department of Social Work and Social Administration, The University of Hong Kong; (3) Center for Social Welfare Studies, Beijing Normal University, Beijing, China |
| Marselia Tan | Care Policy and Evaluation Centre (CPEC), Department of Health Policy, London School of Economics and Political Science, United Kingdom |
| Alisha Vara | University of Auckland, New Zealand |
| Pengcheng Wang | Department of Social Work and Social Administration, The University of Hong Kong, Hong Kong |
| Gloria Wong | (1) Department of Social Work and Social Administration; (2) Sau Po Centre on Ageing, The University of Hong Kong, Hong Kong |

1. The metric combines both the basic activities of daily life and instrumental basic activities of daily life. [↑](#footnote-ref-1)
2. Dementia here refers to any type of dementia, which includes Alzheimer's Disease. [↑](#footnote-ref-2)
3. Detailed Egger’s test results can be found in Table S6. [↑](#footnote-ref-3)
4. Detailed Egger’s test results can be found in Table S6. [↑](#footnote-ref-4)
5. Detailed Egger’s test results can be found in Table S6. [↑](#footnote-ref-5)
6. The study was ultimately excluded as it is identified as a "quasi-experimental study" by the authors themselves, despite indications in the text that some elements of randomisation were involved. [↑](#footnote-ref-6)
7. The study was ultimately excluded as it is said to use a "quasi-experimental design" by the authors themselves, despite indications in the text that some elements of randomisation were involved. [↑](#footnote-ref-7)
8. The study was ultimately excluded as it is said to use a "quasi-experimental design" by the authors themselves, despite indications in the text that some elements of randomisation were involved. [↑](#footnote-ref-8)
